# Identification of genomic regions for grain yield and yield stability and their epistatic interactions

**DOI:** 10.1038/srep41578

**Published:** 2017-02-01

**Authors:** Deepmala Sehgal, Enrique Autrique, Ravi Singh, Marc Ellis, Sukhwinder Singh, Susanne Dreisigacker

**Affiliations:** 1 International Maize and Wheat Improvement Center (CIMMYT), Km. 45, Carretera Méx-Veracruz, El Batán, Texcoco, CP 56237, México

## Abstract

The task of identifying genomic regions conferring yield stability is challenging in any crop and requires large experimental data sets in conjunction with complex analytical approaches. We report findings of a first attempt to identify genomic regions with stable expression and their individual epistatic interactions for grain yield and yield stability in a large elite panel of wheat under multiple environments via a genome wide association mapping (GWAM) approach. Seven hundred and twenty lines were genotyped using genotyping-by-sequencing technology and phenotyped for grain yield and phenological traits. High gene diversity (0.250) and a moderate genetic structure (five groups) in the panel provided an excellent base for GWAM. The mixed linear model and multi-locus mixed model analyses identified key genomic regions on chromosomes 2B, 3A, 4A, 5B, 7A and 7B. Further, significant epistatic interactions were observed among loci with and without main effects that contributed to additional variation of up to 10%. Simple stepwise regression provided the most significant main effect and epistatic markers resulting in up to 20% variation for yield stability and up to 17% gain in yield with the best allelic combination.

The projected global demand for wheat by 2050^1^, coupled with the predicted detrimental effects of climate change on its production^2^,^3^, demands a great push towards developing high yielding varieties with stable yields. Traditional breeding methods have achieved tremendous success in the last century in pushing the annual genetic gain to up to 1% in grain yield, but to cope with 2% yearly increase in world population further efforts are required^4^. It was envisioned a long time ago that the application of molecular markers would strengthen and expedite breeding for complex traits. The real scenario is that even though hundreds of publications have reported quantitative trait loci (QTL) for yield potentially in almost all crops^5^,^6^, marker-assisted selection (MAS) for yield or yield stability has never been realistic. This reinforces the complex genetic architecture of grain yield involving multiple small effect loci, interacting and displaying significant genotype x environment interactions. Attempts to dissect yield QTL using bi-parental populations allowed poor resolution of the QTL due to limited recombination events resulting in the overestimation of QTL effects^7^. The creation of nested association mapping (NAM) and multiparent advanced generation intercrosses (MAGIC) designs, offering a balance of statistical power and mapping resolution, was a great leap forward towards dissection of genetic architecture of complex traits^8^. However, development of such experimental populations is fairly cumbersome, and can be cost and time-consuming.

The advances in next generation sequencing technologies and high-throughput genotyping systems have revolutionized the field of plant genomics, offering a range of modern tools and methods to scientists to characterize their populations. Genotyping-by-sequencing (GBS), which simultaneously performs SNP discovery and genotyping, has taken marker technologies to a next level providing breeders with a cost-effective genome scan of their breeding lines^9^. This has particularly benefited wheat where marker number and density have often been the limiting factors^10^. Therefore, rapidly growing numbers of breeding lines are being genotyped^10^. This has made existing phenotypic data on breeding lines amenable to genome wide association mapping (GWAM), which has become a leading approach for complex trait dissection and identification of novel and superior alleles using existing germplasm.

Since its first application for milling quality in wheat^11^, GWAM studies have been extended to a wider set of traits, mainly those involved in disease resistance^12^. Although a few studies have been piloted for mapping grain yield and yield components in irrigated or stress environments^12^,^13^,^14^,^15^,^16^,^17^,^18^, hitherto no study has reported genomic regions associated with yield stability using this approach. The present study is one of the first efforts in this direction. Yield stability can be defined as the genotype’s ability to perform consistently across a wide range of environments or alternatively the genotype’s ability to interact less with the environments. Several indices have been reported to quantify yield stability. Here, we have quantified yield stability across six contrasting environments using the Lin and Binn’s superiority index^19^ where superiority of cultivar performance is measured by distance mean squares from the maximum responses across all locations (see Materials and methods section for details).

Despite the increasing awareness that epistasis forms the genetic basis of complex traits, the significance of epistasis in the genetics of grain yield has been rarely investigated in a wheat GWAM panel^20^. Unravelling QTL with epistatic effects using a GWAM design will have more potential compared to linkage mapping because fixation of QTL alleles will be less likely in a naturally segregating population, and the power of GWAM will be considerably larger. In the current study, we first identified marker-trait associations (MTA) for grain yield and yield stability that are expressed in multiple environments using a large set of breeding lines from the International Maize and Wheat Improvement Center (CIMMYT). We then investigated the level of the epistatic interactions among them and with loci without main effects. Finally, we performed epistatic scans among all loci without main effects to understand the relevance and magnitude of epistatic interactions for the above mentioned traits. To extract the best out of the association and epistatic scans, we integrated the most stable main effect and epistatic loci into a forward stepwise regression analysis and identified the best marker and allelic combinations conferring highest yield stability. In this way, the current study led to the identification of genetic markers that tag genomic regions, which confer reliable yields across multiple irrigated and stress environments as well as 21 wheat lines that express the beneficial alleles.

## Results and Discussion

### Suitability of the GWAM panel to dissect grain yield and yield stability

Our GWAM panel contained a large (720 entries) set of candidate wheat lines (Supplementary Table S1) from the CIMMYT International Bread Wheat Screening Nursery (IBWSN) in 2012. The panel therefore had the advantage of detecting MTA directly in the relevant current breeding germplasm^11^,^18^,^21^. Previous association studies in wheat have been conducted using gene bank accessions including lines of different geographic origin and adaptions to distinct environments^14^,^22^,^23^. The use of such panels maximizes the genetic diversity and reduces the population structure which is ideal for GWAM, but for quantitative traits such as yield, multiple, small effect MTA are difficult to be directly applied in breeding programs as time consuming and complex gene introgression strategies are required.

A comprehensive phenotypic data set was collected on our panel under multiple irrigated (favorable) and stress environments (mild to severe drought- and heat-stressed environments; see details in Materials and methods section) which allowed us to quantify yield stability by meta-analysis of all environments. Additionally, the size of the panel made the study of multi-locus epistatic interactions feasible as reasonable statistical power could be obtained with such a large experimental data set, the lack of which has often been a limitation in GWAM studies^24^. Our GWAM panel was therefore well-suited for achieving both purposes; identifying markers associated with yield stability and performing epistatic scans.

The panel showed a wide range of variation for grain yield (GY) (Supplementary Table S2), susceptibility indices and yield stability coefficient (Supplementary Table S3). A moderate heritability (*h*^*2*^ = 0.54) was witnessed for grain yield across environments which further raised the scope of obtaining stable MTA. However, days to heading (DH) also showed a wide variation in the panel (Supplementary Table S2) and its correlation with GY was often significant (Supplementary Table S4 and Supplementary Fig. S1); positive correlation in two irrigated environments and negative correlations in stressed low-yielding environments. These results emphasize the effect of “escape” of early lines in determining yield under water and heat stressed conditions. Plant height (PH) was positively associated with GY in most environments. These results implicated that confounding phenological effects need to be taken into account^18^.

### Genetic diversity, population structure and linkage disequilibrium decay

The average gene diversity (Table 1) of the panel was higher than in other sets of cultivars and breeding lines genotyped with SNP markers^25^,^26^,^27^, an excellent base for GWAM research. Gene diversity was, however, slightly lower than in a previously compiled CIMMYT germplasm panel, the Wheat Association Mapping Initiative (WAMI) population (gene diversity = 0.340)^18^. The higher diversity in WAMI panel is probably due to the high frequency (39%) of lines with 1B.1R translocations, and also because relatively diverse synthetic wheat derived lines were included in this population.

**Figure 1:**
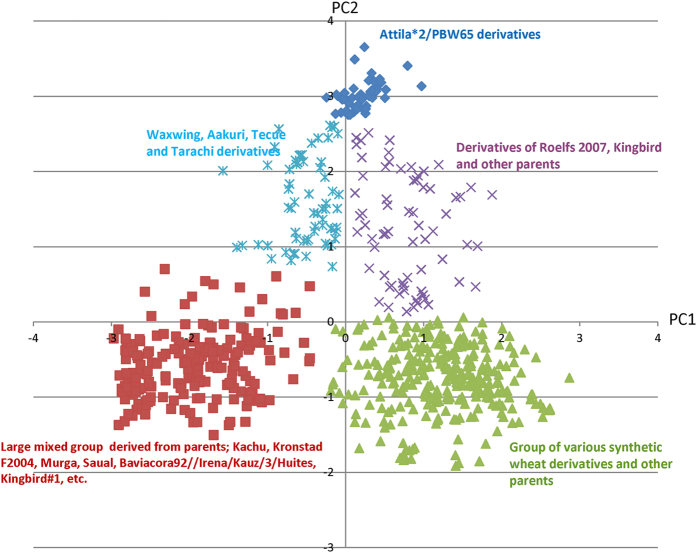
The first and second principal axes from the principal component analysis of C45IBWSN using 6040 GBS SNPs. Each data point represents a genotype. Representative parents or genetic backgrounds for individuals in each of the subgroups are indicated.

The principal component analysis (PCA) showed five groups in the panel (Fig. 1). The first principal component separated a group of synthetic hexaploid wheat derived lines from a second large group with mixed pedigrees including CIMMYT key parents such as Kachu, Kronstad F2004, Murga, Saual, Baviacora 92//Irena/Kauz/3/Huites and Kingbird#1. Lines with Attila*2/PBW65 in their genetic background formed a third group. Two other smaller groups were mainly dominated by lines with key parents Roelfs F2007 and Kingbird, and Waxwing, Akuri, Tecue and Tarachi in their genetic backgrounds, respectively. Since the lines included in the panel were selected based on pedigree information, the partitioning of the genetic diversity in subgroups remained a distinct feature of the panel, similar to what has been observed in other elite wheat collections^13^,^17^,^26^. The presence of non-random “background” co-ancestry among accessions is a common feature of cultivars and advanced breeding materials.

**Figure 2:**
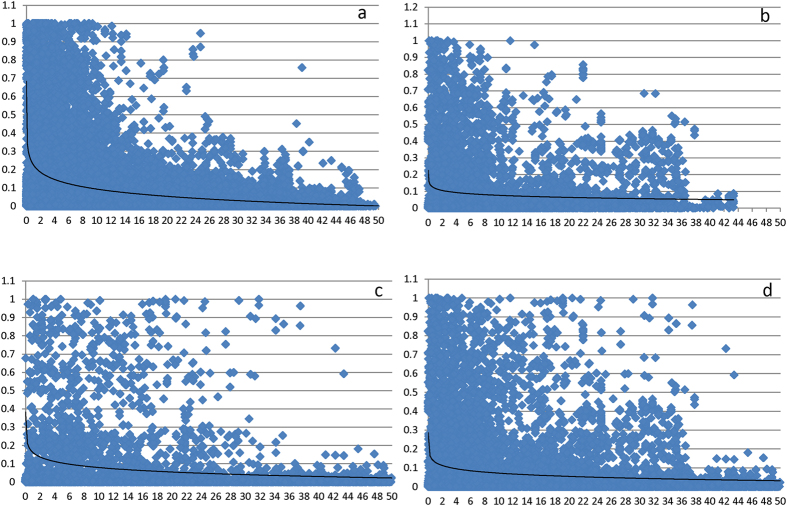
Linkage disequilibrium decay across the A (**a**), B (**b**) and D (**c**) sub-genomes and across the entire genome (**d**) of wheat. The values on the Y-axis represent the squared correlation coefficient *r*^2^ and the values at X-axis represent genetic distance in centiMorgan.

The decay of linkage disequilibrium (LD) over physical/genetic distance in a population determines the density of marker coverage needed to perform GWAM. A faster LD decay indicates that a higher marker density is required to capture the markers close enough to the causal loci^28^. LD in our panel decayed within 10, 4 and 8 cM for A, B and D sub-genomes (Fig. 2a–c), respectively, and for the whole genome, it decayed at 5 cM (Fig. 2d). While the genome-wide LD decay is comparable to the recent studies in wheat^17^,^21^,^26^,^29^, differences were observed at sub-genome levels. In most studies, the D sub-genome showed the slowest LD decay among the three sub-genomes, whereas we observed a faster LD decay in the D sub-genome as compared to the sub-genome A. Faster LD decline in the D sub-genome, compared with A or B sub-genomes, was also observed in the WAMI population^18^ and Chinese germplasm^30^; which could be related to the use of synthetics and wild relatives driving more recombination in the D genome. These results also support the need for higher marker density in the D sub-genome to improve gene localization by GWAM. Alternatively, our results suggested that long range haplotypes in the A sub-genome might have been selected, whether intentionally or unintentionally during the breeding process, thus leading to a slower decay as compared to the D sub-genome.

### Marker-trait associations for GY and yield stability

Since a moderate population structure was witnessed in our panel, a regression analysis was conducted to investigate the effects of population structure on the measured traits which revealed a greater influence on PH (14.2 to 17.3%) and a modest effect on DH (4.1 to 7.0%) and GY (6.3 to 9.2%) (Supplementary Table S5). A mixed linear model (MLM) which took into account PCA, kinship or both for GWAM based on the Bayesian Information Criterion^31^ was therefore used to deal with the confounding effect of the population structure. The use of MLM effectively eliminated the excess of low *P*-values for all traits and indices (see Q-Q plots in Supplementary Figs S2 and S3), when compared with naïve association analysis (no population structure adjustment at all). A comparison of MLM was also made with multi-locus mixed model (MLMM), which took into account multiple loci instead of single loci, for the identification of associations missed using MLM.

Thirty seven MTA for GY, delineating into 27 quantitative trait loci (QTL), were identified considering all environments. Of these, 16 MTA belonging to 11 QTL were shared between at least two or more environments. A comparison with previously published studies is summarized in Supplementary Table S6 revealing that nine QTL identified on chromosomes 1B, 2B, 3A, 4A, 5B, 6A and 7B were novel of which four were shared among multiple environments and five were environment specific. Of the environment-specific novel QTL, three QTL were discovered for heat tolerance on chromosomes 1B and 6A and two QTL on chromosomes 2B and 5B were observed for drought tolerance, providing additional targets for further QTL validation and fine mapping. MLMM identified three additional novel environments specific QTL on chromosomes 1B, 3A and 3B (Supplementary Table S7).

**Figure 3:**
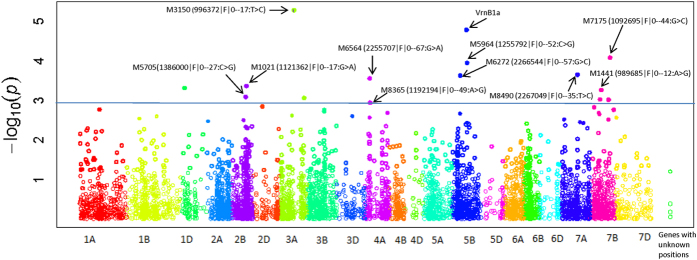
Marker-trait associations (MTAs) identified for yield stability coefficient using 6,040 GBS SNP markers. The MTAs shared with grain yield under different irrigated, water and heat stress environments are shown with arrows.

For yield stability coefficient, 15 MTA delineating into 11 QTL were identified on chromosomes 2B, 3A, 3B, 4A, 5B, 7A and 7B (Table 2). The GBS sequences of these markers are provided in Supplementary Table S8). As expected, identified associations were shared with GY in different environments, drought susceptibility index (DSI) and/or heat susceptibility index (HSI) (Table 2 and Fig. 3). A comparison with previous studies revealed that six QTL coincided with known genomic regions (previous known QTL for GY and yield components) and five QTL were novel (Table 2). The most significant genomic region identified for yield stability coefficient on chromosome 3A at 137.7 cM (associated with GY in all environments) was completely linked with a locus identified for harvest index (HI) under irrigated and drought conditions in the CIMMYT WAMI population^17^ and also coincided with the genomic region reported to be associated with GY and yield components (kernel per spike, thousand kernel weight) in other recent studies^32^,^33^. The second QTL on chromosome 3A at 242.7 cM was novel. A GY QTL reported in a recent GWAM study^15^ was ~24 cM away from this second QTL on chromosome 3A. The MTA identified on chromosome 3B for yield stability coefficient was unique as it was shared with GY only in the drought stress environments and DSI (Table 2). This genomic region, however, has been reported to be associated with GY under irrigated and stress (drought and heat stress) environments in a bi-parental cross^34^.

Two genomic regions were identified on chromosome 4A associated with yield stability coefficient (Table 2), of which the first within a 4.8 cM interval explaining 12% of the variation (identified by markers M6564, M612 and M8365) represents a novel genomic region with almost similar effects on GY in the different environments. The second genomic region on chromosome 4A at 233.7 cM, with higher allelic effects in irrigated environment compared to stress environments, is within 1.8 to 7.4 cM from QTL reported to be associated with GY, DSI and HI on chromosome 4AL^13^,^17^,^35^,^36^. The genomic region identified at 153.6 cM on chromosome 5B most likely falls in the region of the stable QTL for GY identified in three elite populations under irrigated environments by GWAM^15^. The second genomic region between 82.46 and 82.77 cM on chromosome 5B was ~12 to 32 cM away from a QTL identified either for GY in irrigated and drought stress environments^13^,^17^,^34^ or HSIGY^37^ and hence was novel.

Two novel QTL for yield stability were identified on chromosome 7B; the first QTL identified by marker M7175 with higher allelic effects in irrigated and heat stress environments as compared to drought environments is within ~12 and 15 cM from a major QTL identified for heat tolerance^38^ and for grains per spike in 11 environment^39^, respectively, while the second QTL identified by marker M1441 is 14 to 16 cM from a major QTL identified for GY, HI and DSI^36^.

The MTA for DH and PH are summarized in Supplementary Tables S9 and S10. By comparing MTA of DH and PH with that of GY and yield stability coefficient it was observed that many MTA were either common between DH, GY and yield stability coefficient or many markers associated with GY were located within 5 cM of QTLs of DH, suggesting that many genomic regions were confounded with phenology. These confounding effects can be minimized by following one of the two approaches; (a) use DH directly as a co-variable in association analysis or (b) identify the genes associated with variation in flowering time and use their marker scores as co-variables in GWAM^18^. We followed the latter approach and first localized the known major genes controlling DH on our consensus map (Supplementary Table S11) to identify any co-localization of GY and yield stability coefficient QTL with DH genes and then investigated their association with DH, GY and yield stability coefficient. Significant associations of *Vrn-B1a* (dominant spring allele) and *Ppd-D1a* (photoperiod insensitive allele) alleles with DH and GY (Supplementary Tables S6 and S9) were observed, specifically in drought stress environments (Supplementary Fig. S4). Significant association of *Vrn-B1a* with yield stability coefficient (Table 2) was also found, suggesting that the genes *Vrn-B1* and *Ppd-D1* were major sources of variation affecting DH, GY and yield stability in our panel. Hence, a second GWAM was conducted using *Vrn-B1a* and *Ppd-D1a* marker scores as co-variables for GY and only *Vrn-B1a* marker scores as co-variable for yield stability coefficient. For GY, 22 out of 37 MTA and for yield stability 12 out of 15 MTA were significant after the co-variate analysis (Table 2 and Supplementary Table S6).

An important observation was that using MLMM, the *Vrn-D3* gene was revealed to be associated with DH in most environments (Supplementary Table S12), which was missed using MLM thus indicating the increased power of MLMM due to inclusion of multiple causal sites^40^ as compared to the random single marker approach in MLM. Moreover, one additional locus was identified on chromosome 2D, which was significantly associated with DH in two irrigated environments (Supplementary Table S12). This locus might be in LD with the *Ppd-D1* gene, although this needs to be confirmed as we could not map the *Ppd-D1* gene due to scanty representation of GBS markers on chromosome 2D.

### Epistatic interactions and stepwise regression of most significant markers

**Figure 4:**
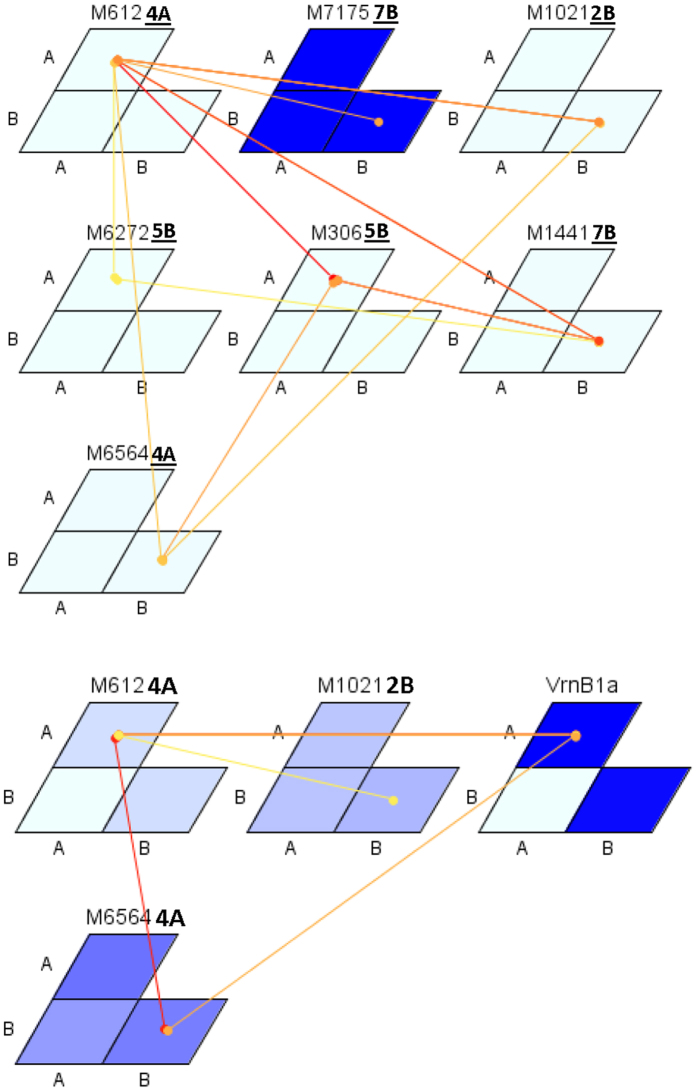
Epistatic interactions for yield stability coefficient without (above) and with (below) major genes included in model. Each matrix indicates one marker. The A and B are the two alleles for each marker. The magnitude of marker effect (F value) is represented with shades of blue (dark blue with stronger interaction). The magnitude of epistatic interaction is presented with colors from yellow to red (stronger interaction).

It is well established that in any GWAM study, even the best associations show only modest R^2^ values for the corresponding SNPs, specifically for complex traits such as those undertaken in the present study. In the literature, this has been termed as “unexplained or missing heritability”^41^ and has been attributed to various factors, with the two most important being the involvement of complex epistatic interactions^41^ and unidentified rare variants^42^. Given the marker density and sample size, the current study was adequately powered to find alleles that are common, but a larger panel coupled with higher density of SNPs would empower us to detect rare variants of small effects. Knowing that we could not run additional association scans for identifying rare variants, we ran exhaustive epistatic scans for GY and yield stability coefficient to study the contribution of epistasis to their genetic architecture and to identify stable epistatic loci to include in forward stepwise regression analysis along with main effect loci to identify the best marker and allelic combinations leading to higher yield stability and mean grain yield. Epistatic interactions were studied in depth; (1) among marker-associated QTLs; (2) among marker-associated QTLs and the major genes controlling DH; (3) among marker-associated QTLs and markers without main effects; and (4) among markers without main effects (Fig. 4, Table 3, Supplementary Figs S5 and S6 and Supplementary Tables S13 and S14). In the first case, 60% of markers associated with GY in different environments were involved in significant (*P* < 0.001) epistatic interactions and so were 50% of the markers associated with yield stability coefficient (Table 3 and Fig. 4). When the genes controlling DH were included in the model (second case), interactions were observed among nine marker-associated QTL for GY and *Vrn-B1* and *Ppd-D1* alleles, and among three marker-associated QTL and *Vrn-B1* for yield stability coefficient (Table 3 and Fig. 4). In the third case, again moderate to strong interactions were observed in all environments, except in one of the irrigated environments (Supplementary Table S13). Additional interactions in the genome were witnessed among markers without main effect, influencing both GY and yield stability coefficient (Supplementary Table S14). In most cases, interactions observed were stronger for yield stability coefficient than for GY. The magnitude of interactions and the frequent involvement of *Vrn-B1* and *Ppd-D1* alleles in interactions with marker-associated QTL for GY and yield stability coefficient confirms the complexities of the genetic control of grain yield and yield stability. This also implies that many QTL must be manipulated simultaneously in order to obtain a significant impact.

**Figure 5:**
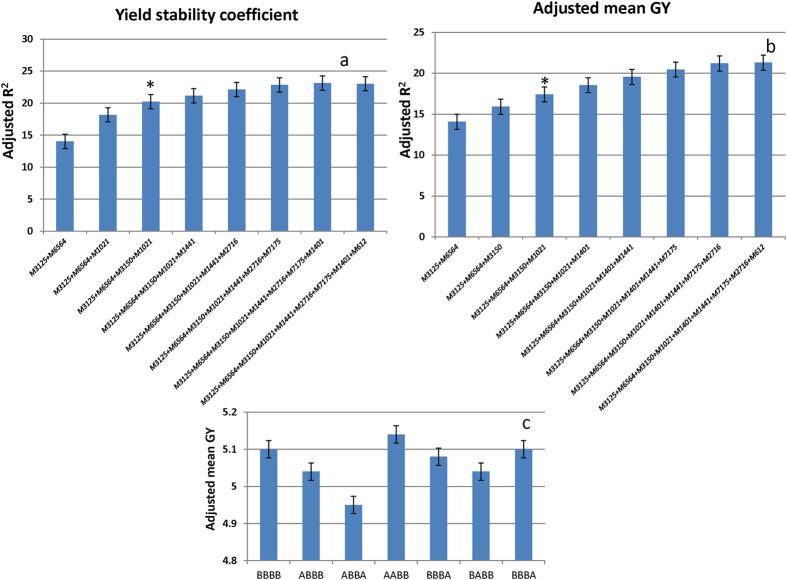
Stepwise regression of the nine markers for (**a**) yield stability coefficient and (**b**) adjusted mean GY across six environments. The starred column in part a of the figure indicates the best marker combination that resulted in highest R^2^ for yield stability and in part b it indicates the R^2^ of the mean GY for the four marker SNP combinations. The best allelic combination resulting in highest mean GY is shown in part c. Lines with ‘A’ calls for the SNPs associated with markers M3125 and M6564 and ‘B’ calls for markers M3150 and M1021 showed the highest mean GY.

To extract the best from our GWAM and epistatic interaction analyses, the nine most stable markers with main and epistatic effects (M6564, M612, M1021, M7175, M1441, M3150, M3125, M2716 and M1401) were selected for a forward stepwise regression analysis to identify possible marker combinations resulting in higher mean GY across environments. The results of the analysis revealed that the combination of four markers (M3125, M6564, M3150 and M1021) resulted in the highest R^2^ (20%) for yield stability coefficient, and including more markers did not result in any further significant increase (Fig. 5a). The R^2^ for mean GY across environments for the same four-marker combination was 17.5% (Fig. 5b). The allele combinations of these four markers resulting in the highest mean GY across environments is presented in Fig. 5c. Twenty one lines possessing this allelic combination have been identified for future trials, of which 18 lines showed mean GY ≥ 5 tons/ha across environments (Supplementary Table S15). Further, their low DSI and HSI (≤1.0) indicate their high tolerance for drought and heat stress, respectively. An *in silico* analysis of these four markers was conducted to identify the candidate genes. Two of these markers showed hits with genes (heavy metal transport/detoxification superfamily protein and DNA J heat shock N-terminal domain-containing protein) having a proven role under stress^43^,^44^ (Supplementary Table S16).

## Conclusion

Here we identified both, known associations (previously reported QTLs for grain yield) and new candidate genomic regions for grain yield and yield stability coefficient. The coincidence of the QTLs for yield stability coefficient in the same genomic regions as QTLs reported for GY under drought and heat stress and/or for DSI and HSI in previous studies, specifically on chromosomes 3A, 3B and 4A, suggests that many “stability-associated genes” could simply be “stress-responsive genes”. We successfully demonstrated a way to use the results of exhaustive association and epistatic scans in molecular breeding of complex traits such as grain yield and yield stability. Combining GWAM and the study of epistasis have clear potential for optimizing molecular marker based selection strategies for GY and yield stability.

## Materials and Methods

### Plant materials and field experiments

The GWAM panel consisted of 720 breeding lines that formed the candidate of the 45^th^ IBWSN in 2012 in CIMMYT’s spring bread wheat program (Supplementary Table S1).

The panel was evaluated at the Borlaug experimental research station in Ciudad Obregon, Mexico, during the 2009–2010 and 2010–2011 growing seasons in six contrasting environments in combination with three management conditions [planting in beds (B), planting on the flat (F) and zero tillage farming (ZT)]. Optimum environments included three well-irrigated treatments with five irrigations in different management conditions [B-5IR 2009–2010 (I), BZT-5IR 2010–2011 (II) and F-5IR 2010–2011 (III)]. The five irrigations were applied at germination and 40, 70, 95 and 115 days after the first irrigation with a total water supply of 550 mm in optimum environments. Stress environments included two drought stress treatments [(B-2IR 2010–2011 (IV) and B-drip 2010–2011(V)] and one high temperature treatment due to a 90 day delay in the planting date [(B-heat 2010–2011 (VI); average T_max_ > 32 °C]. For environment V, soil available water was estimated by collecting samples up to 120 cm at 30 cm interval prior to planting and two irrigations (at germination and 56 days after the first irrigation) were applied with the total amount of water available for the plant equaling 180 mm. For environment IV, two irrigations (at germination and 40 days after the first irrigation) were applied using furrow irrigation with a total water supply of 280 mm. Six irrigations (at germination and 30, 48, 60, 70 and 80 days after the first irrigation) were applied in environment VI using furrow irrigation. The experimental design was an alpha lattice design with three replications. Each experiment included 28 lines and two checks. For each of the trials conducted in beds, the plots comprised of two 0.80 m raised beds of 2.8 meters long with three planting rows on the top of each bed. The ZT management was different from the bed trials in complete absence of tilling for three consecutive years. For the flat plot trial, experimental plots were 1.3 by 4.5 m.

The phenotypic traits evaluated in this study were grain yield (GY), days to heading (DH) and plant height (PH) for all environments except V for which PH was not recorded. DH was recorded as the number of days from planting until 50% of the spikes in each plot had completely emerged above the flag leaves. PH was recorded as the average of three values for each plot measured in centimeter from the soil surface to the tip of the spike excluding awns.

### Phenotypic data analysis

For each environment, the adjusted entry mean of the genotypes were calculated for GY relative to the performance of the two check cultivars Roelfs F2007 and RSM NORMAN F2008 in SAS 9.4 using PROC GLM. The traits for which single replications (DH and PH) were measured, the adjusted mean was calculated by the formula Y = (Y_ij_ − Y_i_) + Y_all trials_, where Y_ij_ is value of the entry for a trial, Y_i_ is mean of checks of that trial and Y_all trials_ is the mean of checks of all trials.

Broad sense heritability (*h*^*2*^) was calculated across environments from variance components obtained from REML analysis in GenStat edition 14^th^ using formula *h*^*2*^ = Vg/((Vg + Vge/n) + (Verr/nr)) where, Vg = genotypic variance, Vge = genotype x environment variance, Verr = error variance, n = number of environments and r = replicates. Pearson phenotypic correlation coefficients among traits were obtained in Minitab 15 (http://www.minitab.com/en-IT/products/minitab/).

A drought and heat susceptibility index (DSI and HSI, respectively) was calculated as described by Fisher and Maurer^45^ and expressed by the relationship for DSI as (1 − *Y*_d_/*Y*_i_)/S, where *Y*_d_ = yield of each line in drought stress environment (B-drip 2010–2011), *Y*_i_ = yield of each line under fully irrigated conditions (II) and S = 1 − (*Y*_dm_/*Y*_im_) where *Y*_dm_ and *Y*_im_ are the mean yield over all genotypes evaluated under drought stress and irrigated conditions, respectively. Similarly, HSI was calculated as (1 − *Y*_h_/*Y*_*i*_)/S, where *Y*_h_ = yield of each line under high temperature conditions (VI), and S = 1 − (*Y*_hm_/*Y*_im_) where *Y*_hm_ is the mean yield over all genotypes evaluated under high temperature conditions. These susceptibility indices indicate tolerance of genotypes under drought and heat stress environments. DSI and HSI values greater than 1 indicate above average susceptibility to drought and heat stress, respectively.

A yield stability coefficient was calculated for all entries using Lin and Binn’s superiority index (*Pi*)^19^ in GenStat edition 14^th^. This index determines the mean squared distance between genotype *i* and the genotype with the maximum yield within each environment as a genotypic superiority measure (*Pi*) with following expression:


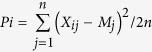


where *Pi* = superiority index of the i-th entry, *X*_*ij*_ = yield of the i-th entry in the j-th environment, *M*_*j*_ = maximum response obtained among all the entries in the j-th environment, and n = number of environments. The genotypes with small *Pi* values considered to have a stable performance across environments.

### Genotyping-by-sequencing

Genomic DNA was extracted from dried leaves collected from five plants per line using a modified CTAB method described in CIMMYT laboratory protocols (http://repository.cimmyt.org/xmlui/handle/10883/3221). Genotyping was accomplished using GBS developed by DArT Pty. Ltd., Yarralumla, Australia. The detailed methodology is described elsewhere^46^. A consensus map version 3.0 (http://www.diversityarrays.com/sequence-maps) was provided as a part of the service which contained ~64 K GBS markers and 4000 DArTs as anchored markers.

### Genotyping with known allele-specific markers associated with major genes controlling flowering time and plant height

The GWAM panel was also genotyped with 22 allele-specific markers (Supplementary Table S17) for major genes controlling vernalization response (*Vrn-1, Vrn-3*), photoperiod sensitivity (*Ppd-1*) and plant height (*Rht-1, Rht-2*). SNP marker genotyping was performed using the LGC Genomics KASP chemistry (www.lgcgenomics.com). The detailed protocols used for PCR reaction mixtures and thermal cycling conditions for amplifying SNP and STS markers are described elsewhere^18^.

### Diversity parameters and linkage disequilibrium

Out of a total of 11,221 GBS SNP markers, 6,040 markers with known map positions, filtered with minor allele frequency >0.05 and missing data points <30% were used for diversity and LD analyses. The two diversity parameters, gene diversity^47^ and polymorphic information content (PIC)^48^, were calculated for sub-genomes and across the whole genome using an in-house script in R. The LD measure *r*^*2*^ (squared correlation coefficient) was calculated among all pairs of loci using TASSEL version 4.0 (http://www.maizegenetics.net) and were plotted against the genetic distance in centiMorgans to determine the pattern of LD decay across the three sub-genomes and whole genome. A cut off of *r*^*2*^ = 0.1 was considered as the critical distance up to which a QTL region extends.

### Genome wide association mapping

GWAM was conducted using two models; the mixed linear model (MLM) in the “EMMAX” (Efficient Mixed Model Association-Expedited) package and multi-locus mixed model (MLMM) designed for mapping complex traits in structured populations^40^. The kinship matrix was calculated by the VanRaden algorithm^49^. The covariance matrix was derived by PCA using the function PRCOMP from the STATS package in R. A forward model selection approach using the Bayesian information criterion (BIC)^31^ was used to select the best model for GWAM of each phenotype. In the case of trait being unaffected by population structure, only kinship matrix representing cryptic relatedness (very common in breeding lines having shared ancestries) was taken in subsequent GWAM analysis while in the other scenario where population structure did affect trait means, appropriate number of PCs were selected based on highest BIC value to include in GWAM along with kinship matrix. The susceptibility indices and yield stability coefficient were treated as traits in GWAM. A false discovery rate (FDR) adjusted at 0.05 was initially used to determine the *P*-values threshold for declaring significant MTA. However, due to high stringency of FDR-adjusted *P*-values and the potential risk of type II error, the criterion of selecting the *P*-values obtained within the bottom 0.1 percentile of the distribution was utilized^50^. Thus, a threshold of P < 0.001 was used to declare significant MTA.

Adjacent co-segregating markers of a MTA were assigned to a unique QTL region upon meeting the following conditions: ≤5 cM of inter-marker genetic distance (based on LD decay across genome) and presence of strong LD among markers (with *r*^*2*^ values ≥ 0.5).

### Assessment of epistatic interactions

The genotypic and phenotypic data, PCA and kinship matrices were used to analyze epistatic interactions. A linear regression model was used to calculate *P* values for two- and three-locus marker interactions for grain yield and yield stability coefficient using an *in house* designed script in R environment (https://www.rproject.org/). A significant threshold of *P* < 0.001 was used to declare significant marker-marker interactions.

### Stepwise regression

To discover the best allelic combination for yield stability and highest yield across environments, a stepwise regression analysis was conducted in GenStat 14^th^ Edition. All possible combinations of the selected markers were utilized and drawn against the adjusted R^2^ for yield stability and mean adjusted grain yield across environments.

### *In-silico* analysis

GBS sequences of the markers were aligned to Synthetic W7984/Opata M85 PopSeq contigs and wheat genome assembly IWGSC1.0 using Bowtie2 version 2.2.2.9^51^. The Variant Effect Predictor (VEP) available on the Ensemble website at http://www.ensembl.org/info/docs/tools/vep/index.html was used to determine the functional consequences of the variant within 5 kb of the SNP location.

## Additional Information

**How to cite this article**: Sehgal, D. *et al*. Identification of genomic regions for grain yield and yield stability and their epistatic interactions. *Sci. Rep.* **7**, 41578; doi: 10.1038/srep41578 (2017).

**Publisher's note:** Springer Nature remains neutral with regard to jurisdictional claims in published maps and institutional affiliations.

## Supplementary Material

Supplementary File

## Figures and Tables

**Table 1:** Gene diversity and polymorphic information content (PIC) of sub-genomes and across genome with 6040 GBS markers.

Sub-genome	Gene-diversity	PIC
A sub-genome	0.31	0.25
B sub-genome	0.27	0.22
D sub-genome	0.18	0.15
Across genome	0.25	0.21

**Table 2:** SNP markers associated (*p* < 0.001) with yield stability coefficient (Yld Sta) and shared with grain yield (GY) under different treatments, drought susceptibility index (DSI) and heat susceptibility index (HSI) using MLM and MLMM analyses.

SNP code	Clone ID	Chr	Pos (cM)	SNP	Yld Sta	GY Bed ZT 5IR		GY Flat 5IR		GY Bed drip		GY Bed 2IR		GY Bed heat		DSI		HSI		Comment
					R^2^	R^2^	All Eff**	R^2^	All Eff	R^2^	All Eff	R^2^	All Eff	R^2^	All Eff	R^2^	All Eff	R^2^	All Eff	
M1021*^,b^	1121362	2B	147.53	G/A	5.2	3.7	−0.183					3.2	−0.146	5.1	−0.223					Known^17^
M5705^a^	1386000	2B	150.25	C/G	4.3	2.9	−0.115			4.4	0.148					4.1	−0.025			
M3150*^,b^	996372	3A	137.76	T/C	4.6	4.2	−0.124	4.6	−0.172	4.2	−0.141	5.6	−0.183	3.5	−0.114	3.5	0.023			Known^17^,^32^,^33^
M7482*^,a^	2265707	3A	242.72	G/C	3.3	3.5	0.089	5.2	0.156			4.5	0.129							Novel
M7810^a^	1098221	3B	297.56	A/G	3.4					3.4	0.101	5.2	0.119			3.3	−0.017			Known^15^,^34^
M6564*^,b^	2255707	4A	48.95	G/A	5.6	5.0	0.180							5.6	0.199			4.5	−0.037	Novel
M612*^,b^	977534	4A	48.95	G/C	3.5	2.2	0.120					3.4	0.141	3.5	0.126					
M8365*^,b^	1192194	4A	53.74	A/G	3.4	4.5	0.148					3.2	0.138	3.7	0.136					
M2195*^,a^	992502	4A	233.75	C/A	4.2	5.3	0.129					4.2	0.063	4.5	0.069					Known^13^,^15^,^17^,^35^
*Vrn-B1a*^b^		5B	165.80		8.1			3.5	−0.170	4.6	−0.157	5.4	−0.231			4.4	0.027			
M5964*^,a^	1255792	5B	153.69	C/G	4.3	3.3	−0.101	2.5	−0.102			3.5	−0.108							Known^13^,^15^
M6272*^,b^	2266544	5B	82.77	G/C	4.2	3.4	−0.121			3.1	−0.089									Novel
M306^a^	976877	5B	82.46	G/A	4.6					4.4	−0.138					4.0	0.024			
M8490*^,a^	2267049	7A	170.91	T/C	3.2	4.5	0.095			2.9	0.074	2.6	0.068							Known^13^,^15^
M7175*^,b^	1092695	7B	202.20	G/C	4.8	3.6	0.143			2.3	0.106			4.4	0.150			3.6	−0.030	Novel
M1441*^,b^	989685	7B	112.24	A/G	3.4			4.1	−0.136	2.4	−0.076									Novel

For each marker, chromosome (Chr) position (Pos), percentage variation (R^2^) and allelic effects (All Eff) are presented. Underlined markers represent the same genomic region on a chromosome. *The asterisk sign represents markers that survived covariate analysis using the *Vrn-B1* gene as covariate. Markers that were identified within 5 cM of known QTL are reported as known and the corresponding reference has been superscripted. For markers belonging to same genomic region/QTL, the comment has been put only on the first marker reported from that genomic region. The sequences of the markers are provided in Supplementary Table S8. The entire set of genotypic data is made available on DATAVERSE (http://hdl.handle.net/11529/10479). ^a,b^Signs in superscript indicate markers identified only in MLM analysis (superscript a) and in both MLM and MLMM analyses (superscript b). **Sign of the allelic effect estimate is with respect to the nucleotide that is second in alphabetical order. From a breeding point of view, environments where positive effects have been reported second allele in alphabetical order is favorable, and where negative effects have been reported, the first allele is favorable. This is best exemplified from marker M5705 where allele ‘G’ is favorable under bed drip conditions and allele ‘C’ is favorable under BedZT 5IR conditions.

**Table 3:** Epistatic interactions for grain yield and yield stability coefficient among associated markers and among associated markers and major genes.

Treatment	DH genes included (+/−)	Locus combination studied	Marker and gene alleles involved in epistasis	Percentage variation explained by interaction (%)
Bed 5IR	−	2	M2118(AA) M4418(AA)	3.0
	−	3	M6533(AA) M2118(AA) M4418(AA)	2.9
	−	3	M4311(BB) M2118(AA) M4418(AA)	3.0
	+	2	M2118(AA) M4418(AA)	3.0
	+	3	M4311(BB) M2118(AA) M4418(AA)	3.0
	+	3	*Ppd-D1a*(AA) M2118(AA) M4418(AA)	3.1
	+	3	*Ppd-D1*Mercia type(BB) M2118(AA) M4418(AA)	3.1
Bed ZT 5IR	−	2	M7175(BB) M8365(AA)	3.6
	−	2	M6564(BB) M1021(BB)	3.5
	−	2	M8365(BB) M1021(BB)	3.6
	−	2	M8365(AA) M2195(AA)	3.5
	−	3	M6564(BB) M8365(BB) M1021(BB)	3.6
	+	2	—	—
	+	3	*Ppd-B1a*(AA) *Vrn-B1a*(BB) M7175(BB)	3.2
Flat 5IR	−	2	M7482(AA) M5994(AA)	4.3
	−	2	M7482(AA) M7629(AA)	4.4
	−	3	M5964(BB) M7482(AA) M3762(BB)	3.6
	−	3	M5964(BB) M7482(AA) M7629(AA)	4.0
	−	3	M7482(AA) M5994(AA) M7629(AA)	4.4
	+	2	—	—
	+	3	—	—
Bed drip	−	2	—	
	−	3	M1441(AA) M4718(BB) M5705(AA)	3.4
	−	3	M1441(BB) M7175(BB) M306(AA)	3.4
	−	3	M7175(BB) M306(AA) M3150(BB)	3.3
	+	2	*Ppd-D1a*(AA) *Vrn-B1a*(AA)	2.9
	+	2	*Ppd-D1*Mercia type(BB) *Vrn-B1a* (AA)	3.3
	+	3	*Ppd-D1*Mercia type(BB) *Ppd-D1a*(AA) *Vrn-B1a* (AA)	3.2
**Treatment**	**Major genes included (+/−)**	**Locus combination studied**	**Marker and gene alleles involved in epistasis**	**Percentage variation explained by interaction (%)**
Bed 2IR	−	2	M612(AA) M1021(BB)	2.6
	−	2	M612(AA) M9969(AA)	2.5
	−	3	M612(AA) M9969(AA) M7810(BB)	2.5
	+	2	*Vrn-B1a*(AA) M612(AA)	2.7
	+	3	*Vrn-B1a* (AA) M612(AA) M1021(BB)	2.3
Bed heat	−	2	M612(AA) M4129(BB)	3.2
	−	2	M3989(AA) M4129(BB)	3.0
	−	2	M4129(BB) M6564(BB)	3.5
	−	3	M612(AA) M3989(AA) M4129(BB)	3.0
	−	3	M3989(AA) M4129(BB) M6564(BB)	3.1
	+	2	M612(AA) M4129(BB)	3.2
	+	2	M3989(AA) M4129(BB)	3.0
Yield stability	+	2	M4129(BB) M6564(BB)	3.5
	+	3	M612(AA) M3989(AA) M4129(BB)	3.0
	+	3	M3989(AA) M4129(BB) M6564(BB)	3.1
	−	2	M1441(BB) M612(AA)	9.3
	−	2	M612(AA) M1021(BB)	9.0
	−	3	M1441(BB) M306(AA) M612(AA)	9.2
	−	3	M1441(BB) M306(AA) M6564(BB)	9.3
	−	3	M1441(BB) M612(AA) M1021(BB)	9.2
	−	3	M7175(BB) M612(AA) M1021(BB)	8.9
	−	3	M612(AA) M6564(BB) M1021(BB)	9.3
	+	2	*Vrn-B1a* (AA) M612(AA)	8.6
	+	2	*Vrn-B1a* (AA) M6564(BB)	8.6
	+	3	*Vrn-B1a* (AA) M612(AA) M6564(BB)	8.5
	+	3	*Vrn-B1a* (AA) M612(AA) M1021(BB)	8.5

Underlined markers represent main epistatic locus interacting with other marker associated QTL.
