# Supplementary material for: Identification of genomic regions for grain yield and yield stability and their epistatic interactions
Source: Sci Rep. 2017 Feb 1;7:41578. doi: 10.1038/srep41578 (PMC5286416; doi:10.1038/srep41578)
Supplement: Supplementary File [file srep41578-s1.pdf]

**Genome wide association study for wide environmental adaptation in a large spring bread wheat population using genotyping-by-sequencing approach**

Deepmala Sehgal[1], Enrique Autrique[1], Ravi Singh[1], Marc Ellis[1], Sukhwinder Singh[1], Susanne Dreisigacker[1]*

[1]International Center for Maize and Wheat Improvement (CIMMYT), Km. 45, Carretera Méx-Veracruz, El Batán, Texcoco, México, CP 56237

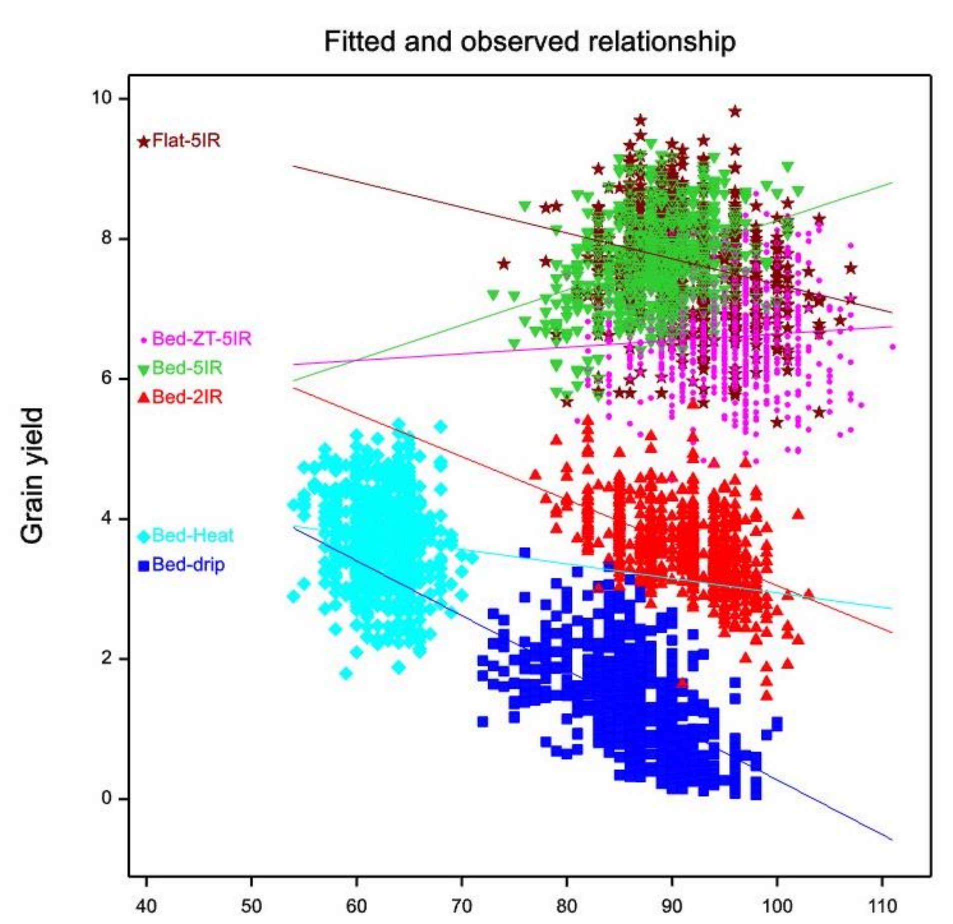


Supplementary Fig. S1 Phenotypic correlations between grain yield and days to heading under different environments

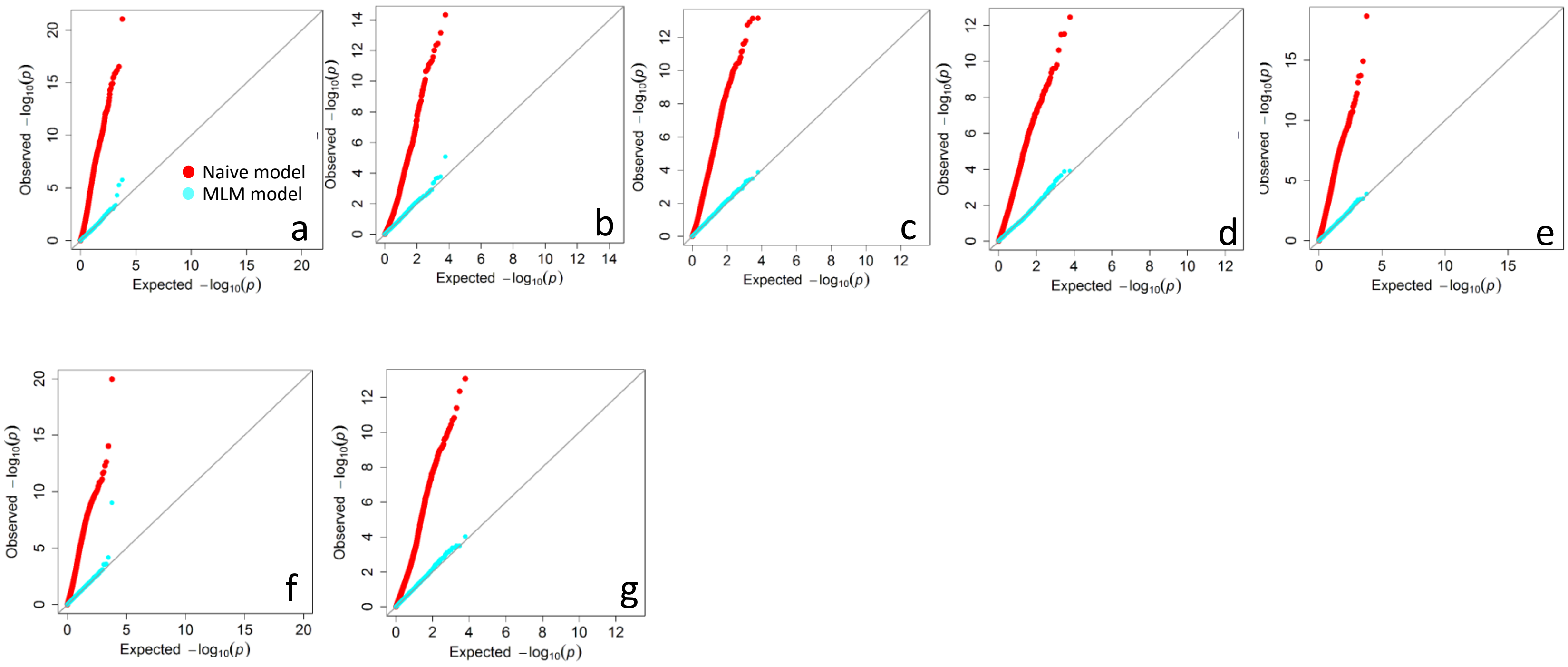


Supplementary Fig. S2 Quantile – Quantile plots for naïve and mixed linear model (using PC, kinship or PC + kinship based on BIC (Bayesian Information Content) for yield stability (a) and grain yield under different environments (b; Bed 5IR, c; Flat 5IR, d; Bed ZT 5IR, e; Bed drip, f; Bed 2IR and g; Bed heat)

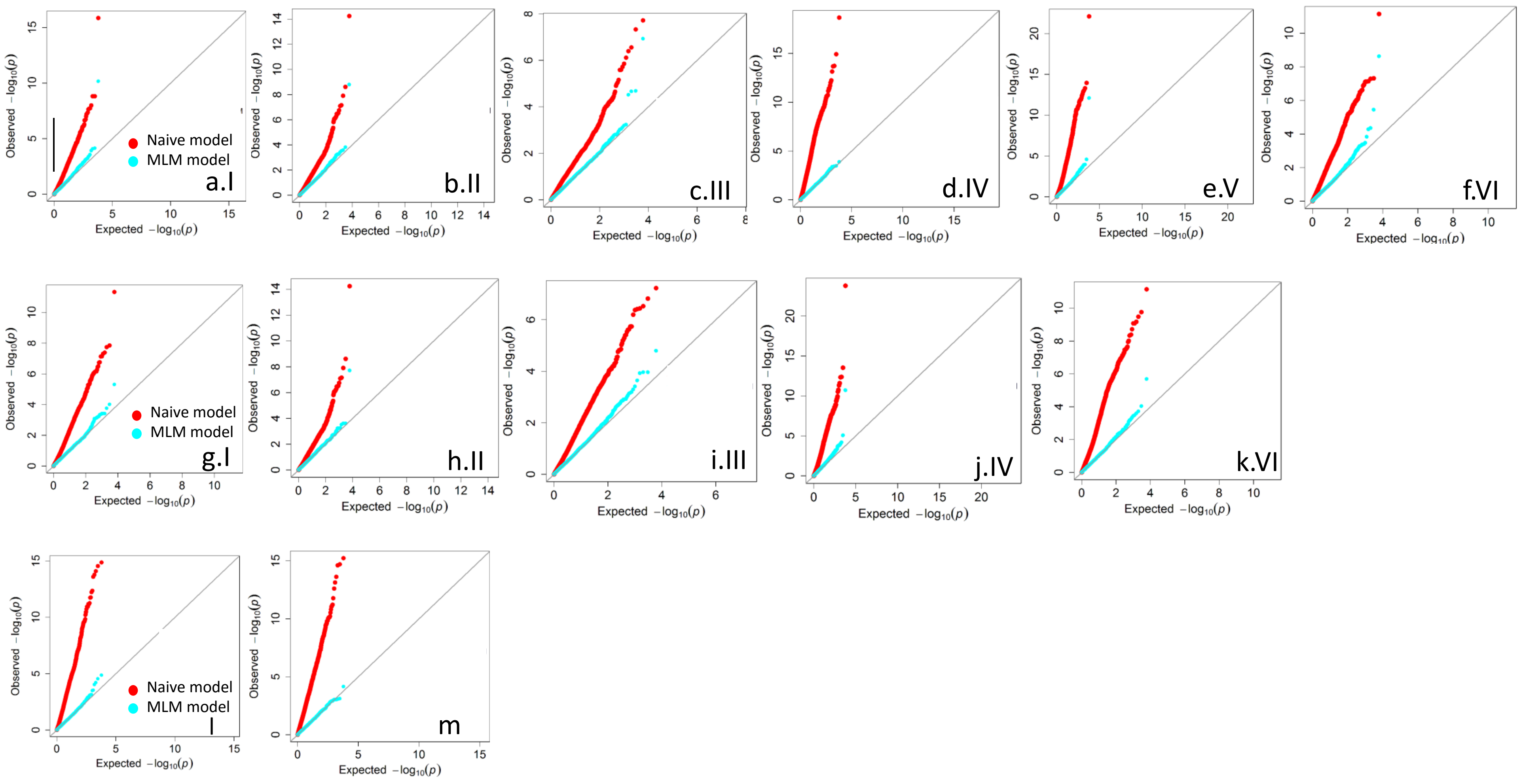


Supplementary Fig. S3 Quantile – Quantile plots for naïve and mixed linear model for days to heading (a-f) and plant height (g-k) in different environments (I; Bed 5IR, II; Bed ZT 5IR, III; Flat 5IR, IV; Bed 2IR, V; Bed drip, and VI; Bed heat) and for DSI (l) and HSI (m)

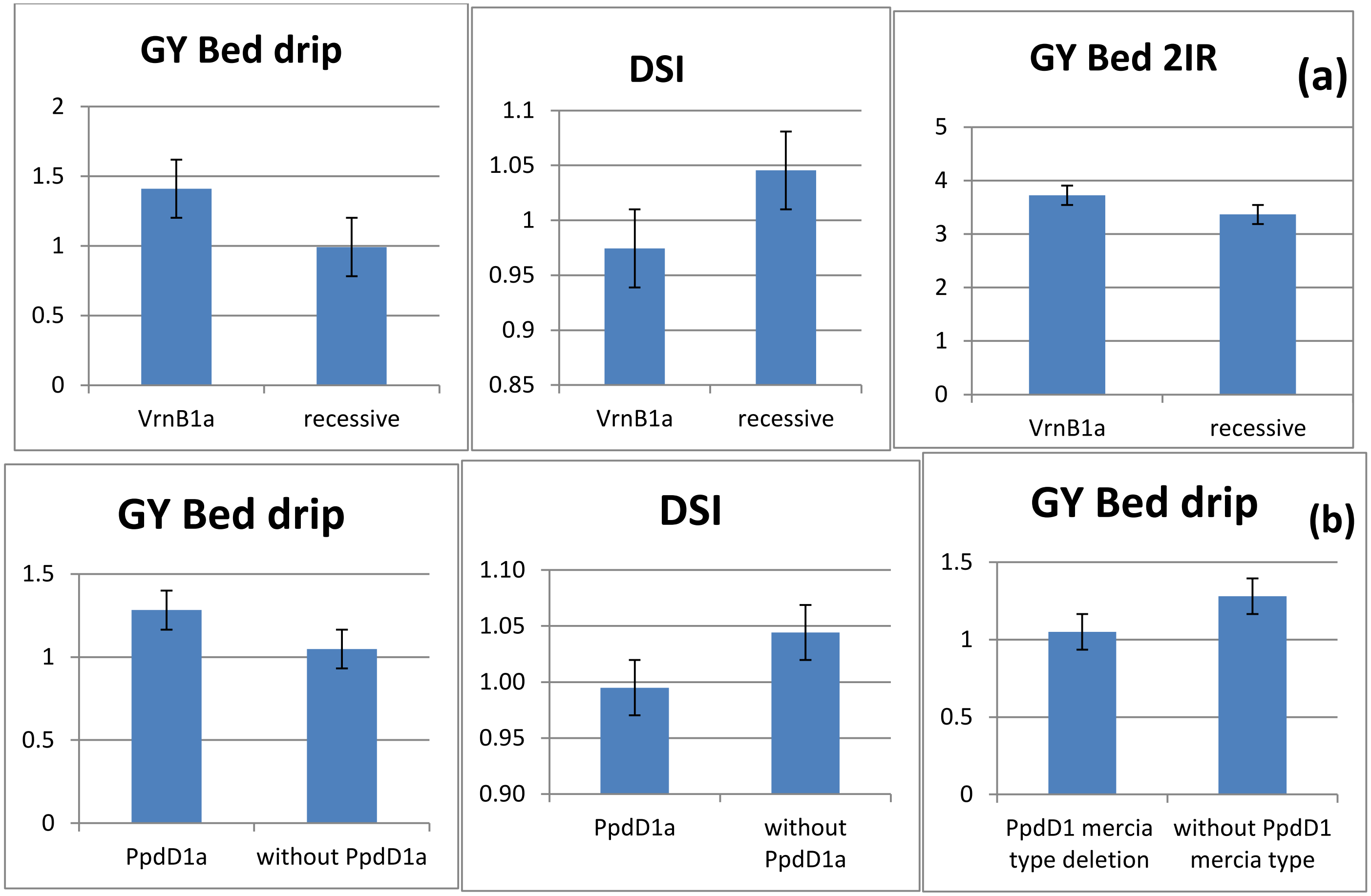


Supplementary Fig. S4 Allelic effects of *Vrn-B1a* on grain yield (GY) under both drought stress environments and drought susceptibility index (DSI) (a) and allelic effects of *Ppd-D1a* (Ciano type deletion conferring photoperiod insensitivity) and *Ppd-D1* Mercia type insertion conferring photoperiod sensitivity on GY under severe drought stress treatment and DSI. Earlier flowering lines carrying dominant spring allele *Vrn-B1a* or photoperiod insensitive allele *Ppd-D1a* are higher yielding under drought stress environments with lower DSI than lines carrying recessive *vrn* allele or photoperiod sensitive allele (Mercia type insertion).

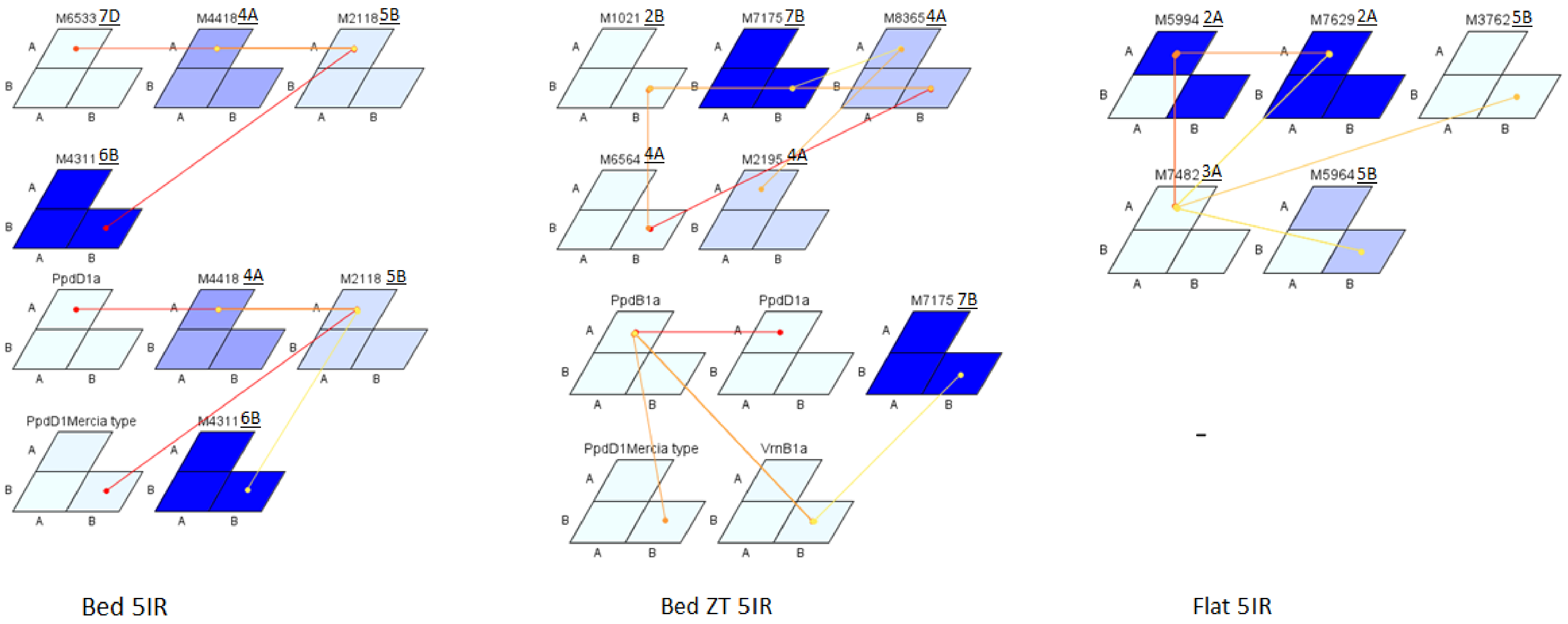


Supplementary Fig. S5 For each of the irrigated conditions, epistatic interactions are shown among associated markers without (above) and with major genes (below) included in the model. The A and B are the two alleles for each marker. The magnitude of marker effect (F value) is represented with shades of blue from light to dark (higher effect). The magnitude of epistatic interaction is presented with colors from yellow to red (stronger interaction).

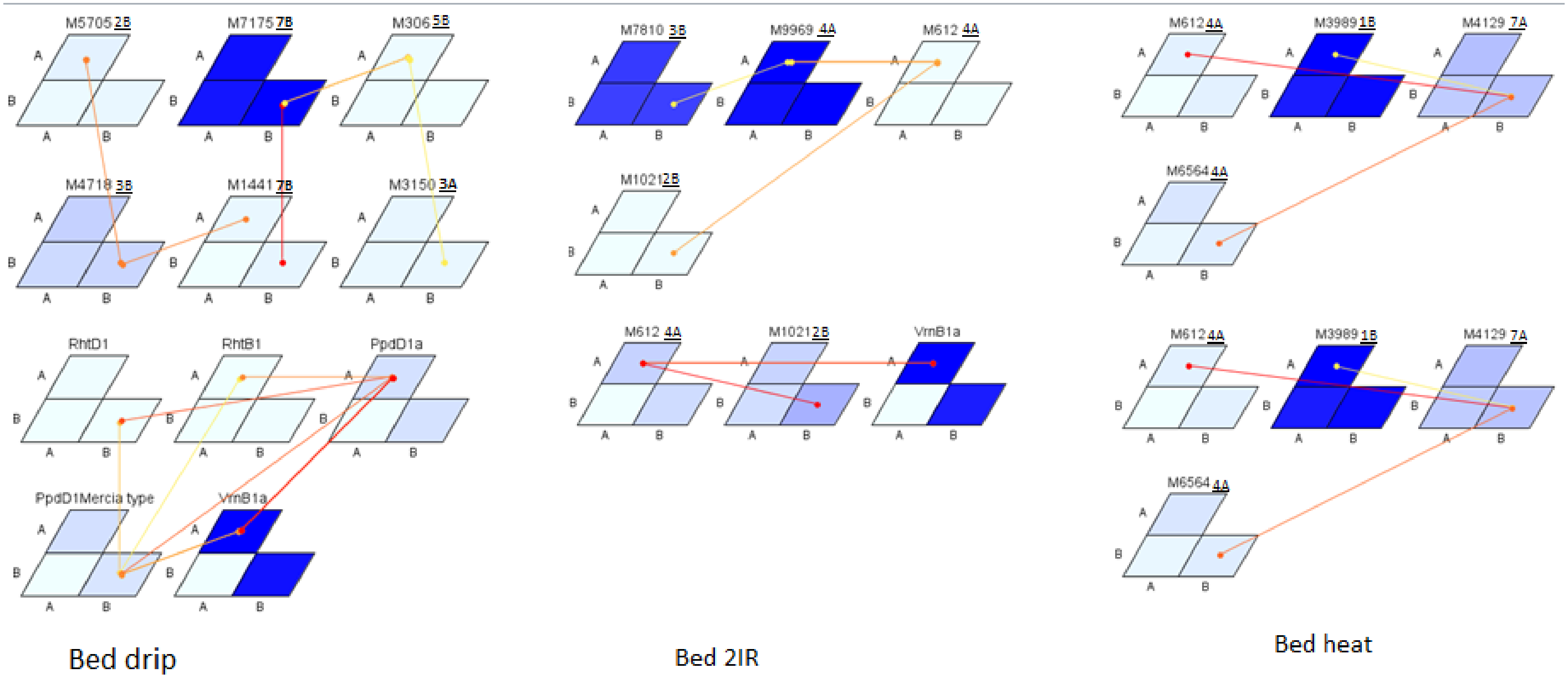


Supplementary Fig. S6 For each of the stressed conditions, epistatic interactions are shown without (above) and with major genes (below) included in the model. Each matrix indicates one marker. The A and B are the two alleles for each marker. The magnitude of marker effect (F value) is represented with shades of blue. The magnitude of epistatic interaction is presented with colors from yellow to red (stronger interaction).

Supplementary Table S1 Genotypes used in the present study

| S. No. | GID | Cross Name |
|---|---|---|
| 1 | 4785245 | GAN/AE.SQUARROSA (236)//CETA/AE.SQUARROSA (895)/3/MAIZ/4/INQALAB 91 |
| 2 | 5866000 | MEX94.27.1.20/3/SOKOLL//ATTILA/3*BCN |
| 3 | 5893304 | SERI/BAV92//WBLL1 |
| 4 | 5893342 | PUB94.15.1.12/FRTL |
| 5 | 5895861 | PASTOR//HXL7573/2*BAU/3/WBLL1 |
| 6 | 5895868 | SERI/BAV92//WBLL1 |
| 7 | 6085788 | QUAIU #1 |
| 8 | 6085793 | QUAIU #1 |
| 9 | 6085804 | KIRITATI//HUW234+LR34/PRINIA |
| 10 | 6174798 | MILAN/S87230//BAV92/3/ROLF07 |
| 11 | 6174802 | SAUAL/KACHU |
| 12 | 6174807 | SAUAL/KACHU |
| 13 | 6174819 | CNO79//PF70354/MUS/3/PASTOR/4/BAV92/5/TACUPETO F2001*2/BRAMBLING |
| 14 | 6174820 | CNO79//PF70354/MUS/3/PASTOR/4/BAV92/5/TACUPETO F2001*2/BRAMBLING |
| 15 | 6174828 | TACUPETO F2001/3/FRET2/WBLL1//TACUPETO F2001 |
| 16 | 6174847 | TACUPETO F2001*2/BRAMBLING//WBLL1*2/BRAMBLING |
| 17 | 6174858 | FRANCOLIN #1/WBLL1 |
| 18 | 6174859 | FRANCOLIN #1/WBLL1 |
| 19 | 6174860 | FRANCOLIN #1//WBLL1*2/BRAMBLING |
| 20 | 6174867 | WBLL1*2/BRAMBLING/5/WBLL1*2/4/SNI/TRAP#1/3/KAUZ*2/TRAP//KAUZ |
| 21 | 6174868 | WBLL1*2/BRAMBLING/5/WBLL1*2/4/SNI/TRAP#1/3/KAUZ*2/TRAP//KAUZ |
| 22 | 6174876 | WBLL1*2/BRAMBLING//KACHU |
| 23 | 6174877 | WBLL1*2/4/SNI/TRAP#1/3/KAUZ*2/TRAP//KAUZ/5/WAXWING/4/SNI/TRAP#1/3/KAUZ*2/TRAP//KAUZ |
| 24 | 6174880 | BECARD/KACHU |
| 25 | 6174882 | BECARD/KACHU |
| 26 | 6174884 | BECARD/KACHU |
| 27 | 6174886 | BECARD/KACHU |
| 28 | 6174887 | BECARD/KACHU |
| 29 | 6174889 | BECARD/KACHU |
| 30 | 6174890 | BECARD/KACHU |
| 31 | 6174891 | FRANCOLIN #1//WBLL1*2/KIRITATI |
| 32 | 6174892 | FRANCOLIN #1//WBLL1*2/KURUKU |
| 33 | 6174895 | ALTAR 84/AE.SQUARROSA (221)//3*BORL95/3/URES/JUN//KAUZ/4/WBLL1/5/MILAN/S87230//BAV92 |
| 34 | 6174896 | ALTAR 84/AE.SQUARROSA (221)//3*BORL95/3/URES/JUN//KAUZ/4/WBLL1/5/MILAN/S87230//BAV92 |
| 35 | 6174901 | ALTAR 84/AE.SQUARROSA (221)//3*BORL95/3/URES/JUN//KAUZ/4/WBLL1/5/MILAN/S87230//BAV92 |
| 36 | 6174903 | ALTAR 84/AE.SQUARROSA (221)//3*BORL95/3/URES/JUN//KAUZ/4/WBLL1/5/MILAN/S87230//BAV92 |
| 37 | 6174904 | ALTAR 84/AE.SQUARROSA (221)//3*BORL95/3/URES/JUN//KAUZ/4/WBLL1/5/MILAN/S87230//BAV92 |
| 38 | 6174926 | WBLL1*2/BRAMBLING//HEILO |
| 39 | 6174927 | WBLL1*2/BRAMBLING//FN/2*PASTOR |
| 40 | 6174949 | QUAIU #3//MILAN/AMSEL |

| | | |
|---|---|---|
| 41 | 6174951 | QUAIU #3//MILAN/AMSEL |
| 42 | 6174952 | QUAIU #3//MILAN/AMSEL |
| 43 | 6174993 | ATTILA*2/PBW65*2//TOBA97/PASTOR |
| 44 | 6175002 | ATTILA*2/PBW65*2//KRONSTAD F2004 |
| 45 | 6175005 | ATTILA*2/PBW65*2//KRONSTAD F2004 |
| 46 | 6175009 | KAUZ/PASTOR//PBW343/3/FRET2/KURUKU//FRET2/4/WBLL1*2/BRAMBLING |
| 47 | 6175010 | KAUZ/PASTOR//PBW343/3/FRET2/KURUKU//FRET2/4/WBLL1*2/BRAMBLING |
| 48 | 6175022 | ROLF07*2/5/REH/HARE//2*BCN/3/CROC_1/AE.SQUARROSA (213)//PGO/4/HUITES |
| 49 | 6175024 | TACUPETO F2001/BRAMBLING*2//KACHU |
| 50 | 6175025 | TACUPETO F2001/BRAMBLING*2//KACHU |
| 51 | 6175030 | TACUPETO F2001/BRAMBLING*2//KACHU |
| 52 | 6175035 | TACUPETO F2001/BRAMBLING*2//KACHU |
| 53 | 6175036 | TACUPETO F2001/BRAMBLING*2//KACHU |
| 54 | 6175038 | TACUPETO F2001/BRAMBLING*2//KACHU |
| 55 | 6175042 | WBLL1*2/KKTS/3/FRET2/TUKURU//FRET2/4/WBLL1*2/KURUKU |
| 56 | 6175044 | WBLL1*2/KURUKU/3/FRET2/TUKURU//FRET2/4/WBLL1*2/KURUKU |
| 57 | 6175046 | WBLL1*2/KURUKU/3/FRET2/TUKURU//FRET2/4/WBLL1*2/KURUKU |
| 58 | 6175048 | WBLL1*2/KURUKU/3/FRET2/TUKURU//FRET2/4/WBLL1*2/KURUKU |
| 59 | 6175056 | WBLL1*2/TUKURU//SAUAL/3/WBLL1*2/BRAMBLING |
| 60 | 6175057 | WBLL1*2/VIVITSI//PRINIA/PASTOR/3/WBLL1*2/BRAMBLING |
| 61 | 6175063 | NAC/TH.AC//3*PVN/3/MIRLO/BUC/4/2*PASTOR/5/KACHU/6/KACHU |
| 62 | 6175067 | NAC/TH.AC//3*PVN/3/MIRLO/BUC/4/2*PASTOR/5/KACHU/6/KACHU |
| 63 | 6175076 | NAC/TH.AC//3*PVN/3/MIRLO/BUC/4/2*PASTOR/5/KACHU/6/KACHU |
| 64 | 6175078 | SAUAL/5/REH/HARE//2*BCN/3/CROC_1/AE.SQUARROSA (213)//PGO/4/HUITES/6/KACHU |
| 65 | 6175127 | SITE/MO//PASTOR/3/TILHI/4/WAXWING/KIRITATI/5/PBW343*2/TUKURU |
| 66 | 6175135 | ALTAR 84/AE.SQUARROSA (221)//3*BORL95/3/URES/JUN//KAUZ/4/WBLL1*2/5/REH/HARE//2*BCN/3/CROC_1/AE.SQUARROSA (213)//PGO/4/HUITES |
| 67 | 6175171 | YAV_3/SCO//JO69/CRA/3/YAV79/4/AE.SQUARROSA (498)/5/LINE 1073/6/KAUZ*2/4/CAR//KAL/BB/3/NAC/5/KAUZ/7/KRONSTAD F2004/8/KAUZ/PASTOR//PBW343 |
| 68 | 6175172 | YAV_3/SCO//JO69/CRA/3/YAV79/4/AE.SQUARROSA (498)/5/LINE 1073/6/KAUZ*2/4/CAR//KAL/BB/3/NAC/5/KAUZ/7/KRONSTAD F2004/8/KAUZ/PASTOR//PBW343 |
| 69 | 6175173 | YAV_3/SCO//JO69/CRA/3/YAV79/4/AE.SQUARROSA (498)/5/LINE 1073/6/KAUZ*2/4/CAR//KAL/BB/3/NAC/5/KAUZ/7/KRONSTAD F2004/8/KAUZ/PASTOR//PBW343 |
| 70 | 6175174 | YAV_3/SCO//JO69/CRA/3/YAV79/4/AE.SQUARROSA (498)/5/LINE 1073/6/KAUZ*2/4/CAR//KAL/BB/3/NAC/5/KAUZ/7/KRONSTAD F2004/8/KAUZ/PASTOR//PBW343 |
| 71 | 6175175 | YAV_3/SCO//JO69/CRA/3/YAV79/4/AE.SQUARROSA (498)/5/LINE 1073/6/KAUZ*2/4/CAR//KAL/BB/3/NAC/5/KAUZ/7/KRONSTAD F2004/8/KAUZ/PASTOR//PBW343 |
| 72 | 6175176 | YAV_3/SCO//JO69/CRA/3/YAV79/4/AE.SQUARROSA (498)/5/LINE 1073/6/KAUZ*2/4/CAR//KAL/BB/3/NAC/5/KAUZ/7/KRONSTAD F2004/8/KAUZ/PASTOR//PBW343 |
| 73 | 6175178 | YAV_3/SCO//JO69/CRA/3/YAV79/4/AE.SQUARROSA (498)/5/LINE 1073/6/KAUZ*2/4/CAR//KAL/BB/3/NAC/5/KAUZ/7/KRONSTAD F2004/8/KAUZ/PASTOR//PBW343 |
| 74 | 6175181 | WAXWING/KIRITATI//FINSI/3/FRANCOLIN #1 |
| 75 | 6175202 | ATTILA*2/PBW65*2//MURGA |
| 76 | 6175205 | ATTILA*2/PBW65*2//MURGA |
| 77 | 6175208 | ATTILA*2/PBW65*2/5/REH/HARE//2*BCN/3/CROC_1/AE.SQUARROSA (213)//PGO/4/HUITES |
| 78 | 6175209 | ATTILA*2/PBW65*2/5/REH/HARE//2*BCN/3/CROC_1/AE.SQUARROSA (213)//PGO/4/HUITES |
| 79 | 6175210 | ATTILA*2/PBW65*2/5/REH/HARE//2*BCN/3/CROC_1/AE.SQUARROSA (213)//PGO/4/HUITES |
| 80 | 6175211 | ATTILA*2/PBW65*2/5/REH/HARE//2*BCN/3/CROC_1/AE.SQUARROSA (213)//PGO/4/HUITES |

| | | |
|---|---|---|
| 81 | 6175212 | ATTILA*2/PBW65*2/5/REH/HARE//2*BCN/3/CROC_1/AE.SQUARROSA (213)//PGO/4/HUITES |
| 82 | 6175213 | ATTILA*2/PBW65*2/5/REH/HARE//2*BCN/3/CROC_1/AE.SQUARROSA (213)//PGO/4/HUITES |
| 83 | 6175216 | WAXWING/4/BL 1496/MILAN/3/CROC_1/AE.SQUARROSA (205)//KAUZ/5/FRNCLN |
| 84 | 6175229 | TOBA97/ATTILA/4/CROC_1/AE.SQUARROSA (205)//KAUZ/3/2*PJN/BOW//OPATA/5/THELIN/3/BABAX/LR42//BABAX/4/BABAX/LR42//BABAX |
| 85 | 6175232 | WBLL1*2/KURUKU/6/CNDO/R143//ENTE/MEXI_2/3/AEGILOPS SQUARROSA (TAUS)/4/WEAVER/5/2*JANZ/7/WBLL1*2/KURUKU |
| 86 | 6175233 | WBLL1*2/KURUKU/6/CNDO/R143//ENTE/MEXI_2/3/AEGILOPS SQUARROSA (TAUS)/4/WEAVER/5/2*JANZ/7/WBLL1*2/KURUKU |
| 87 | 6175234 | WBLL1*2/KURUKU/6/CNDO/R143//ENTE/MEXI_2/3/AEGILOPS SQUARROSA (TAUS)/4/WEAVER/5/2*JANZ/7/WBLL1*2/KURUKU |
| 88 | 6175235 | WBLL1*2/KURUKU/6/CNDO/R143//ENTE/MEXI_2/3/AEGILOPS SQUARROSA (TAUS)/4/WEAVER/5/2*JANZ/7/WBLL1*2/KURUKU |
| 89 | 6175268 | HUW468*2//TOBA97/PASTOR |
| 90 | 6175279 | PBW343*2/KHVAKI*2/4/CROC_1/AE.SQUARROSA (205)//KAUZ/3/2*KAUZ*2/YACO//KAUZ |
| 91 | 6175280 | PBW343*2/KHVAKI*2/4/CROC_1/AE.SQUARROSA (205)//KAUZ/3/2*KAUZ*2/YACO//KAUZ |
| 92 | 6175284 | PBW343*2/KUKUNA*2/4/CROC_1/AE.SQUARROSA (205)//BORL95/3/2*MILAN |
| 93 | 6175296 | UP2338*2/4/SNI/TRAP#1/3/KAUZ*2/TRAP//KAUZ/5/2*WAXWING/4/SNI/TRAP#1/3/KAUZ*2/TRAP//KAUZ |
| 94 | 6175297 | UP2338*2/4/SNI/TRAP#1/3/KAUZ*2/TRAP//KAUZ/5/2*WAXWING/4/SNI/TRAP#1/3/KAUZ*2/TRAP//KAUZ |
| 95 | 6175301 | UP2338*2/4/SNI/TRAP#1/3/KAUZ*2/TRAP//KAUZ/5/2*WAXWING/4/SNI/TRAP#1/3/KAUZ*2/TRAP//KAUZ |
| 96 | 6175310 | SERI.1B*2/3/KAUZ*2/BOW//KAUZ*2/5/REH/HARE//2*BCN/3/CROC_1/AE.SQUARROSA (213)//PGO/4/HUITES |
| 97 | 6175312 | TUKURU//BAV92/RAYON*2/7/YAV_3/SCO//JO69/CRA/3/YAV79/4/AE.SQUARROSA (498)/5/LINE 1073/6/KAUZ*2/4/CAR//KAL/BB/3/NAC/5/KAUZ |
| 98 | 6175345 | TACUPETO F2001/6/OASIS/5*BORL95/5/CNDO/R143//ENTE/MEXI75/3/AE.SQ/4/2*OCI/7/ROLF07 |
| 99 | 6175354 | WAXWING/VORB//FRANCOLIN #1 |
| 100 | 6175358 | BAV92//IRENA/KAUZ/3/HUITES*2/4/VORB |
| 101 | 6175359 | WBLL1*2/VIVITSI//VORB/3/WBLL1*2/BRAMBLING |
| 102 | 6175361 | WBLL1*2/VIVITSI//VORB/3/WBLL1*2/BRAMBLING |
| 103 | 6175382 | NG8675/CBRD//FN/2*PASTOR/4/THELIN/3/2*BABAX/LR42//BABAX |
| 104 | 6175409 | WAXWING*2/HEILO |
| 105 | 6175410 | WAXWING*2/HEILO |
| 106 | 6175411 | WAXWING*2/HEILO |
| 107 | 6175412 | WAXWING*2/HEILO |
| 108 | 6175413 | WAXWING*2/HEILO |
| 109 | 6175417 | WAXWING*2/HEILO |
| 110 | 6175431 | TOBA97/ATTILA//GONDO/3/FRANCOLIN #1 |
| 111 | 6175439 | BAV92//IRENA/KAUZ/3/HUITES/4/GONDO/TNMU/5/BAV92//IRENA/KAUZ/3/HUITES |
| 112 | 6175440 | BAV92//IRENA/KAUZ/3/HUITES/4/GONDO/TNMU/5/BAV92//IRENA/KAUZ/3/HUITES |
| 113 | 6175442 | BAV92//IRENA/KAUZ/3/HUITES/4/GONDO/TNMU/5/BAV92//IRENA/KAUZ/3/HUITES |
| 114 | 6175444 | BAV92//IRENA/KAUZ/3/HUITES/4/GONDO/TNMU/5/BAV92//IRENA/KAUZ/3/HUITES |
| 115 | 6175454 | WBLL1*2/4/YACO/PBW65/3/KAUZ*2/TRAP//KAUZ/5/GONDO/6/WBLL1*2/4/SNI/TRAP#1/3/KAUZ*2/TRAP//KAUZ |
| 116 | 6175471 | WBLL1*2/KURUKU//HEILO/3/WBLL1*2/KURUKU |
| 117 | 6175474 | WBLL1*2/KURUKU//HEILO/3/WBLL1*2/KURUKU |
| 118 | 6175491 | SSERI1/CHIBIA/4/BAV92//IRENA/KAUZ/3/HUITES |
| 119 | 6175493 | SSERI1/CHIBIA/4/BAV92//IRENA/KAUZ/3/HUITES |
| 120 | 6175497 | TUKURU//BAV92/RAYON/3/KRONSTAD F2004 |
| 121 | 6175500 | ATTILA*2/PBW65//WBLL1*2/VIVITSI |

| | | |
|---|---|---|
| 122 | 6175531 | FRET2/TUKURU//FRET2/3/KRONSTAD F2004 |
| 123 | 6175571 | TACUPETO F2001/BRAMBLING//KACHU |
| 124 | 6175573 | WBLL1/MUU #1 |
| 125 | 6175606 | WBLL1/3/STAR//KAUZ/STAR/4/BAV92/RAYON/5/TRAP#1/BOW/3/VEE/PJN//2*TUI/4/BAV92/RAYON |
| 126 | 6175608 | WBLL1/3/STAR//KAUZ/STAR/4/BAV92/RAYON/5/TRAP#1/BOW/3/VEE/PJN//2*TUI/4/BAV92/RAYON |
| 127 | 6175609 | WBLL1/3/STAR//KAUZ/STAR/4/BAV92/RAYON/5/TRAP#1/BOW/3/VEE/PJN//2*TUI/4/BAV92/RAYON |
| 128 | 6175610 | WBLL1/3/STAR//KAUZ/STAR/4/BAV92/RAYON/5/TRAP#1/BOW/3/VEE/PJN//2*TUI/4/BAV92/RAYON |
| 129 | 6175651 | SAUAL/KRONSTAD F2004 |
| 130 | 6175652 | SAUAL/KRONSTAD F2004 |
| 131 | 6175653 | SAUAL/KRONSTAD F2004 |
| 132 | 6175657 | PRINIA/PASTOR/3/PFAU/WEAVER*2//BRAMBLING |
| 133 | 6175659 | PRINIA/PASTOR/3/PFAU/WEAVER*2//BRAMBLING |
| 134 | 6175660 | PRINIA/PASTOR/3/PFAU/WEAVER*2//BRAMBLING |
| 135 | 6175662 | SITE/MO//PASTOR/3/TILHI/4/WAXWING/KIRITATI |
| 136 | 6175667 | ALTAR 84/AE.SQUARROSA (221)//3*BORL95/3/URES/JUN//KAUZ/4/WBLL1/5/REH/HARE//2*BCN/3/CROC_1/AE.SQUARROSA (213)//PGO/4/HUITES |
| 137 | 6175668 | ALTAR 84/AE.SQUARROSA (221)//3*BORL95/3/URES/JUN//KAUZ/4/WBLL1/5/REH/HARE//2*BCN/3/CROC_1/AE.SQUARROSA (213)//PGO/4/HUITES |
| 138 | 6175669 | ALTAR 84/AE.SQUARROSA (221)//3*BORL95/3/URES/JUN//KAUZ/4/WBLL1/5/REH/HARE//2*BCN/3/CROC_1/AE.SQUARROSA (213)//PGO/4/HUITES |
| 139 | 6175679 | MURGA//WAXWING/KIRITATI |
| 140 | 6175680 | MURGA//WAXWING/KIRITATI |
| 141 | 6175694 | MURGA/KRONSTAD F2004 |
| 142 | 6175697 | MURGA/KRONSTAD F2004 |
| 143 | 6175704 | MURGA/KRONSTAD F2004 |
| 144 | 6175705 | MURGA/KRONSTAD F2004 |
| 145 | 6175707 | MURGA/KRONSTAD F2004 |
| 146 | 6175708 | MURGA/KRONSTAD F2004 |
| 147 | 6175717 | REH/HARE//2*BCN/3/CROC_1/AE.SQUARROSA (213)//PGO/4/HUITES/5/KRONSTAD F2004 |
| 148 | 6175718 | REH/HARE//2*BCN/3/CROC_1/AE.SQUARROSA (213)//PGO/4/HUITES/5/KRONSTAD F2004 |
| 149 | 6175720 | REH/HARE//2*BCN/3/CROC_1/AE.SQUARROSA (213)//PGO/4/HUITES/5/KRONSTAD F2004 |
| 150 | 6175721 | REH/HARE//2*BCN/3/CROC_1/AE.SQUARROSA (213)//PGO/4/HUITES/5/KRONSTAD F2004 |
| 151 | 6175740 | ATTILA*2/PBW65//MURGA |
| 152 | 6175744 | ATTILA*2/PBW65//MURGA |
| 153 | 6175757 | BAV92//IRENA/KAUZ/3/HUITES/6/ALD/CEP75630//CEP75234/PT7219/3/BUC/BJY/4/CBRD/5/TNMU/PF85487 |
| 154 | 6175758 | BAV92//IRENA/KAUZ/3/HUITES/6/ALD/CEP75630//CEP75234/PT7219/3/BUC/BJY/4/CBRD/5/TNMU/PF85487 |
| 155 | 6175897 | WBLL1*2/CHAPIO//HEILO |
| 156 | 6175899 | WBLL1*2/CHAPIO//HEILO |
| 157 | 6175900 | WBLL1*2/CHAPIO//HEILO |
| 158 | 6175902 | WBLL1*2/CHAPIO//HEILO |
| 159 | 6175930 | WBLL1*2/KURUKU//HEILO |
| 160 | 6175941 | WBLL1*2/KURUKU//HEILO |
| 161 | 6175947 | WBLL1*2/KURUKU//HEILO |

| | | |
|---|---|---|
| 162 | 6175958 | ATTILA*2/PBW65*2//KACHU |
| 163 | 6175962 | ATTILA*2/PBW65*2//KACHU |
| 164 | 6175963 | ATTILA*2/PBW65*2//KACHU |
| 165 | 6175970 | ATTILA*2/PBW65*2//MESIA |
| 166 | 6175974 | ATTILA*2/PBW65*2/4/CROC_1/AE.SQUARROSA (205)//BORL95/3/2*MILAN |
| 167 | 6175976 | ATTILA*2/PBW65*2/4/CROC_1/AE.SQUARROSA (205)//BORL95/3/2*MILAN |
| 168 | 6175979 | ATTILA*2/PBW65*2/4/CROC_1/AE.SQUARROSA (205)//BORL95/3/2*MILAN |
| 169 | 6175982 | ATTILA*2/PBW65*2/4/CROC_1/AE.SQUARROSA (205)//BORL95/3/2*MILAN |
| 170 | 6175983 | ATTILA*2/PBW65*2/4/CROC_1/AE.SQUARROSA (205)//BORL95/3/2*MILAN |
| 171 | 6175986 | ATTILA*2/PBW65*2/4/CROC_1/AE.SQUARROSA (205)//BORL95/3/2*MILAN |
| 172 | 6175989 | WAXWING*2/4/BOW/NKT//CBRD/3/CBRD |
| 173 | 6175997 | BABAX/LR42//BABAX/3/BABAX/LR42//BABAX/4/PRINIA/PASTOR//HUITES/5/FRET2/WBLL1//TACUPETO F2001 |
| 174 | 6176013 | ROLF07/4/BOW/NKT//CBRD/3/CBRD/5/FRET2/TUKURU//FRET2 |
| 175 | 6176017 | ROLF07*2/4/BOW/NKT//CBRD/3/CBRD |
| 176 | 6176021 | ROLF07*2/3/PRINIA/PASTOR//HUITES |
| 177 | 6176022 | ROLF07*2/3/PRINIA/PASTOR//HUITES |
| 178 | 6176023 | ROLF07*2/4/CROC_1/AE.SQUARROSA (205)//BORL95/3/2*MILAN |
| 179 | 6176024 | ROLF07*2/4/CROC_1/AE.SQUARROSA (205)//BORL95/3/2*MILAN |
| 180 | 6176031 | WBLL1*2/4/SNI/TRAP#1/3/KAUZ*2/TRAP//KAUZ/5/PFAU/WEAVER//BRAMBLING/6/BAV92//IRENA/KAUZ/3/HUITES |
| 181 | 6176039 | WBLL1*2/4/YACO/PBW65/3/KAUZ*2/TRAP//KAUZ*2/6/NG8201/KAUZ/4/SHA7//PRL/VEE#6/3/FASAN/5/MILAN/KAUZ |
| 182 | 6176043 | WBLL1*2/KKTS*2//MILAN/AMSEL |
| 183 | 6176045 | WBLL1*2/KUKUNA/5/PSN/BOW//SERI/3/MILAN/4/ATTILA/6/WBLL1*2/KKTS |
| 184 | 6176047 | WBLL1*2/KUKUNA/5/PSN/BOW//SERI/3/MILAN/4/ATTILA/6/WBLL1*2/KKTS |
| 185 | 6176049 | WBLL1*2/KUKUNA/5/PSN/BOW//SERI/3/MILAN/4/ATTILA/6/WBLL1*2/KKTS |
| 186 | 6176050 | WBLL1*2/TUKURU*2/4/CROC_1/AE.SQUARROSA (205)//BORL95/3/2*MILAN |
| 187 | 6176051 | WBLL1*2/TUKURU*2/4/CROC_1/AE.SQUARROSA (205)//BORL95/3/2*MILAN |
| 188 | 6176054 | WBLL1*2/TUKURU*2/4/CROC_1/AE.SQUARROSA (205)//BORL95/3/2*MILAN |
| 189 | 6176056 | WBLL1*2/TUKURU*2/4/CROC_1/AE.SQUARROSA (205)//BORL95/3/2*MILAN |
| 190 | 6176134 | BAV92//IRENA/KAUZ/3/HUITES*2/4/TNMU |
| 191 | 6176139 | BAV92//IRENA/KAUZ/3/HUITES*2/4/TNMU |
| 192 | 6176149 | NORM/WBLL1//WBLL1/3/TNMU/4/WBLL1*2/TUKURU |
| 193 | 6176173 | UP2338*2/4/SNI/TRAP#1/3/KAUZ*2/TRAP//KAUZ/5/MILAN/KAUZ//CHIL/CHUM18/6/UP2338*2/4/SNI/TRAP#1/3/KAUZ*2/TRAP//KAUZ |
| 194 | 6176174 | UP2338*2/4/SNI/TRAP#1/3/KAUZ*2/TRAP//KAUZ/5/MILAN/KAUZ//CHIL/CHUM18/6/UP2338*2/4/SNI/TRAP#1/3/KAUZ*2/TRAP//KAUZ |
| 195 | 6176178 | UP2338*2/4/SNI/TRAP#1/3/KAUZ*2/TRAP//KAUZ/5/MILAN/KAUZ//CHIL/CHUM18/6/UP2338*2/4/SNI/TRAP#1/3/KAUZ*2/TRAP//KAUZ |
| 196 | 6176187 | WAXWING/2*ROLF07 |
| 197 | 6176189 | WAXWING/2*ROLF07 |
| 198 | 6176196 | BAV92//IRENA/KAUZ/3/HUITES/4/2*ROLF07 |
| 199 | 6176208 | BAV92//IRENA/KAUZ/3/HUITES/4/2*ROLF07 |
| 200 | 6176213 | BAV92//IRENA/KAUZ/3/HUITES/4/2*ROLF07 |
| 201 | 6176224 | FRET2/TUKURU//FRET2/3/MUNIA/CHTO//AMSEL/4/FRET2/TUKURU//FRET2 |
| 202 | 6176225 | FRET2/TUKURU//FRET2/3/MUNIA/CHTO//AMSEL/4/FRET2/TUKURU//FRET2 |

| | | |
|---|---|---|
| 203 | 6176228 | FRET2/TUKURU//FRET2/3/MUNIA/CHTO//AMSEL/4/FRET2/TUKURU//FRET2 |
| 204 | 6176233 | ROLF07*2/KACHU |
| 205 | 6176235 | ROLF07*2/KACHU |
| 206 | 6176292 | ATTILA*2/PBW65*2//MURGA |
| 207 | 6176296 | ATTILA*2/PBW65*2//MURGA |
| 208 | 6176298 | ATTILA*2/PBW65*2//MURGA |
| 209 | 6176303 | ATTILA*2/PBW65*2//MURGA |
| 210 | 6176308 | ATTILA*2/PBW65*2//MURGA |
| 211 | 6176328 | BAV92//IRENA/KAUZ/3/HUITES*2/4/MURGA |
| 212 | 6176329 | BAV92//IRENA/KAUZ/3/HUITES*2/4/MURGA |
| 213 | 6176330 | BAV92//IRENA/KAUZ/3/HUITES*2/4/MURGA |
| 214 | 6176332 | BAV92//IRENA/KAUZ/3/HUITES*2/4/MURGA |
| 215 | 6176334 | ROLF07*2/5/REH/HARE//2*BCN/3/CROC_1/AE.SQUARROSA (213)//PGO/4/HUITES |
| 216 | 6176335 | WBLL1*2/CHAPIO*2//MURGA |
| 217 | 6176336 | WBLL1*2/CHAPIO*2//MURGA |
| 218 | 6176339 | WBLL1*2/CHAPIO*2//MURGA |
| 219 | 6176341 | WBLL1*2/CHAPIO*2//MURGA |
| 220 | 6176344 | WBLL1*2/CHAPIO*2//MURGA |
| 221 | 6176346 | WBLL1*2/KURUKU*2/5/REH/HARE//2*BCN/3/CROC_1/AE.SQUARROSA (213)//PGO/4/HUITES |
| 222 | 6176355 | WBLL1*2/KURUKU*2/5/REH/HARE//2*BCN/3/CROC_1/AE.SQUARROSA (213)//PGO/4/HUITES |
| 223 | 6176359 | WBLL1/FRET2//PASTOR*2/3/MURGA |
| 224 | 6176360 | WBLL1/FRET2//PASTOR*2/3/MURGA |
| 225 | 6176361 | WBLL1/FRET2//PASTOR*2/3/MURGA |
| 226 | 6176368 | KACHU/4/CROC_1/AE.SQUARROSA (205)//BORL95/3/2*MILAN/5/KACHU |
| 227 | 6176372 | KACHU/4/CROC_1/AE.SQUARROSA (205)//BORL95/3/2*MILAN/5/KACHU |
| 228 | 6176395 | KACHU/5/REH/HARE//2*BCN/3/CROC_1/AE.SQUARROSA (213)//PGO/4/HUITES/6/KACHU |
| 229 | 6176403 | SAUAL/4/CROC_1/AE.SQUARROSA (205)//BORL95/3/2*MILAN/5/SAUAL |
| 230 | 6176409 | ATTILA*2/PBW65*2//W485/HD29 |
| 231 | 6176410 | ATTILA*2/PBW65*2//W485/HD29 |
| 232 | 6176414 | ATTILA*2/PBW65*2//W485/HD29 |
| 233 | 6176421 | WAXWING/KIRITATI*2//W485/HD29 |
| 234 | 6176422 | BAV92//IRENA/KAUZ/3/HUITES*2/4/CROC_1/AE.SQUARROSA (224)//KULIN/3/WESTONIA |
| 235 | 6176423 | BAV92//IRENA/KAUZ/3/HUITES*2/4/CROC_1/AE.SQUARROSA (224)//KULIN/3/WESTONIA |
| 236 | 6176424 | BAV92//IRENA/KAUZ/3/HUITES*2/4/CROC_1/AE.SQUARROSA (224)//KULIN/3/WESTONIA |
| 237 | 6176425 | BAV92//IRENA/KAUZ/3/HUITES*2/4/CROC_1/AE.SQUARROSA (224)//KULIN/3/WESTONIA |
| 238 | 6176426 | BAV92//IRENA/KAUZ/3/HUITES*2/4/CROC_1/AE.SQUARROSA (224)//KULIN/3/WESTONIA |
| 239 | 6176427 | BAV92//IRENA/KAUZ/3/HUITES*2/4/CROC_1/AE.SQUARROSA (224)//KULIN/3/WESTONIA |
| 240 | 6176428 | BAV92//IRENA/KAUZ/3/HUITES*2/4/CROC_1/AE.SQUARROSA (224)//KULIN/3/WESTONIA |
| 241 | 6176429 | BAV92//IRENA/KAUZ/3/HUITES*2/4/CROC_1/AE.SQUARROSA (224)//KULIN/3/WESTONIA |
| 242 | 6176431 | ROLF07*2/4/CROC_1/AE.SQUARROSA (224)//KULIN/3/WESTONIA |
| 243 | 6176455 | KACHU*2/3/CHUM18/BORL95//CBRD |
| 244 | 6176472 | KACHU/4/CROC_1/AE.SQUARROSA (205)//KAUZ/3/SASIA/5/KACHU |
| 245 | 6176474 | KACHU/4/CROC_1/AE.SQUARROSA (205)//KAUZ/3/SASIA/5/KACHU |

| | | |
|---|---|---|
| 246 | 6176480 | KACHU/4/CROC_1/AE.SQUARROSA (205)//KAUZ/3/SASIA/5/KACHU |
| 247 | 6176482 | KACHU/4/CROC_1/AE.SQUARROSA (205)//KAUZ/3/SASIA/5/KACHU |
| 248 | 6176487 | KACHU/4/CROC_1/AE.SQUARROSA (205)//KAUZ/3/SASIA/5/KACHU |
| 249 | 6176492 | KACHU/4/CROC_1/AE.SQUARROSA (205)//KAUZ/3/SASIA/5/KACHU |
| 250 | 6176502 | SAUAL*2/6/CNDO/R143//ENTE/MEXI_2/3/AEGILOPS SQUARROSA (TAUS)/4/WEAVER/5/2*PASTOR |
| 251 | 6176506 | SAUAL*2/6/CNDO/R143//ENTE/MEXI_2/3/AEGILOPS SQUARROSA (TAUS)/4/WEAVER/5/2*PASTOR |
| 252 | 6176509 | SAUAL*2/6/CNDO/R143//ENTE/MEXI_2/3/AEGILOPS SQUARROSA (TAUS)/4/WEAVER/5/2*PASTOR |
| 253 | 6176522 | SAUAL/4/CROC_1/AE.SQUARROSA (205)//KAUZ/3/ATTILA/5/SAUAL |
| 254 | 6176523 | SAUAL/4/CROC_1/AE.SQUARROSA (205)//KAUZ/3/ATTILA/5/SAUAL |
| 255 | 6176527 | SAUAL/4/CROC_1/AE.SQUARROSA (205)//KAUZ/3/ATTILA/5/SAUAL |
| 256 | 6176533 | SAUAL/4/CROC_1/AE.SQUARROSA (205)//KAUZ/3/ATTILA/5/SAUAL |
| 257 | 6176537 | SAUAL/4/CROC_1/AE.SQUARROSA (205)//KAUZ/3/ATTILA/5/SAUAL |
| 258 | 6176548 | ATTILA*2/PBW65*2/4/BOW/NKT//CBRD/3/CBRD |
| 259 | 6176551 | ATTILA*2/PBW65*2/4/BOW/NKT//CBRD/3/CBRD |
| 260 | 6176553 | ATTILA*2/PBW65*2/4/BOW/NKT//CBRD/3/CBRD |
| 261 | 6176554 | ATTILA*2/PBW65*2/4/BOW/NKT//CBRD/3/CBRD |
| 262 | 6176556 | ATTILA*2/PBW65*2/4/BOW/NKT//CBRD/3/CBRD |
| 263 | 6176557 | ATTILA*2/PBW65*2/4/BOW/NKT//CBRD/3/CBRD |
| 264 | 6176558 | ATTILA*2/PBW65*2/4/BOW/NKT//CBRD/3/CBRD |
| 265 | 6176559 | ATTILA*2/PBW65*2/4/BOW/NKT//CBRD/3/CBRD |
| 266 | 6176581 | BAV92//IRENA/KAUZ/3/HUITES*2/4/CHIL/CHUM18 |
| 267 | 6176582 | BAV92//IRENA/KAUZ/3/HUITES/4/FN/2*PASTOR/5/BAV92//IRENA/KAUZ/3/HUITES |
| 268 | 6176583 | BAV92//IRENA/KAUZ/3/HUITES/4/FN/2*PASTOR/5/BAV92//IRENA/KAUZ/3/HUITES |
| 269 | 6176584 | BAV92//IRENA/KAUZ/3/HUITES/4/FN/2*PASTOR/5/BAV92//IRENA/KAUZ/3/HUITES |
| 270 | 6176588 | BAV92//IRENA/KAUZ/3/HUITES/4/FN/2*PASTOR/5/BAV92//IRENA/KAUZ/3/HUITES |
| 271 | 6176590 | BAV92//IRENA/KAUZ/3/HUITES*2/4/GONDO/TNMU |
| 272 | 6176591 | BAV92//IRENA/KAUZ/3/HUITES*2/4/GONDO/TNMU |
| 273 | 6176593 | BAV92//IRENA/KAUZ/3/HUITES*2/4/GONDO/TNMU |
| 274 | 6176600 | BAV92//IRENA/KAUZ/3/HUITES*2/4/GONDO/TNMU |
| 275 | 6176601 | BAV92//IRENA/KAUZ/3/HUITES*2/4/GONDO/TNMU |
| 276 | 6176611 | ROLF07*2/4/BOW/NKT//CBRD/3/CBRD |
| 277 | 6176631 | WBLL1/4/HD2281/TRAP#1/3/KAUZ*2/TRAP//KAUZ/5/TACUPETO F2001*2/6/NING MAI 9558 |
| 278 | 6176638 | WBLL1/KUKUNA//TACUPETO F2001*2/3/SHA3/SERI//SHA4/LIRA |
| 279 | 6176639 | WBLL1/KUKUNA//TACUPETO F2001*2/3/SHA3/SERI//SHA4/LIRA |
| 280 | 6176640 | WBLL1/KUKUNA//TACUPETO F2001*2/3/SHA3/SERI//SHA4/LIRA |
| 281 | 6176641 | WBLL1/KUKUNA//TACUPETO F2001*2/3/SHA3/SERI//SHA4/LIRA |
| 282 | 6176647 | WBLL1*2/4/YACO/PBW65/3/KAUZ*2/TRAP//KAUZ*2/5/GONDO |
| 283 | 6176652 | WBLL1*2/KURUKU*2//TNMU |
| 284 | 6176653 | WBLL1*2/KURUKU*2//TNMU |
| 285 | 6176689 | PFAU/WEAVER*2/4/BOW/NKT//CBRD/3/CBRD |
| 286 | 6176696 | PFAU/WEAVER*2//BRAMBLING/3/KAUZ//TRAP#1/BOW/4/PFAU/WEAVER*2//BRAMBLING |
| 287 | 6176697 | PFAU/WEAVER*2//BRAMBLING/3/KAUZ//TRAP#1/BOW/4/PFAU/WEAVER*2//BRAMBLING |
| 288 | 6176730 | SAUAL//CHIL/CHUM18/3/SAUAL |

| | | |
|---|---|---|
| 289 | 6176747 | SAUAL/3/MAYOOR//TK SN1081/AE.SQUARROSA (222)/4/SAUAL |
| 290 | 6176752 | SAUAL/3/MAYOOR//TK SN1081/AE.SQUARROSA (222)/4/SAUAL |
| 291 | 6176829 | SAUAL/3/ACHTAR*3//KANZ/KS85-8-4/4/SAUAL |
| 292 | 6176841 | PBW343*2/KHVAKI*2/3/ACHTAR*3//KANZ/KS85-8-5 |
| 293 | 6176848 | BAV92//IRENA/KAUZ/3/HUITES*2/4/YUNMAI 47 |
| 294 | 6176849 | BAV92//IRENA/KAUZ/3/HUITES*2/4/YUNMAI 47 |
| 295 | 6176866 | WBLL1/KUKUNA//TACUPETO F2001*2/3/CHUANMAI 107 |
| 296 | 6176870 | WBLL1*2/4/YACO/PBW65/3/KAUZ*2/TRAP//KAUZ*2/5/DEMAI 4 |
| 297 | 6176887 | KACHU/YUNMAI 47//KACHU |
| 298 | 6176903 | WBLL1*2/5/CNO79//PF70354/MUS/3/PASTOR/4/BAV92 |
| 299 | 6176904 | WBLL1*2/5/CNO79//PF70354/MUS/3/PASTOR/4/BAV92 |
| 300 | 6176906 | MUNAL #1/FRANCOLIN #1 |
| 301 | 6176912 | MUNAL #1/FRANCOLIN #1 |
| 302 | 6176914 | MUNAL #1/FRANCOLIN #1 |
| 303 | 6176924 | PAURAQ/3/KIRITATI//PRL/2*PASTOR |
| 304 | 6176929 | PFAU/SERI.1B//AMAD/3/WAXWING/4/KIRITATI//PRL/2*PASTOR |
| 305 | 6176982 | CNO79//PF70354/MUS/3/PASTOR/4/BAV92/5/WBLL1*2/BRAMBLING |
| 306 | 6176986 | CNO79//PF70354/MUS/3/PASTOR/4/BAV92/5/WBLL1*2/BRAMBLING |
| 307 | 6176988 | CNO79//PF70354/MUS/3/PASTOR/4/BAV92/5/WBLL1*2/BRAMBLING |
| 308 | 6176995 | QUAIU #1/3/MILAN/S87230//BAV92 |
| 309 | 6177005 | WBLL1*2/BRAMBLING/5/BABAX/LR42//BABAX*2/4/SNI/TRAP#1/3/KAUZ*2/TRAP//KAUZ |
| 310 | 6177010 | WBLL1*2/BRAMBLING/5/BABAX/LR42//BABAX*2/4/SNI/TRAP#1/3/KAUZ*2/TRAP//KAUZ |
| 311 | 6177014 | WBLL1*2/BRAMBLING/5/BABAX/LR42//BABAX*2/4/SNI/TRAP#1/3/KAUZ*2/TRAP//KAUZ |
| 312 | 6177015 | WBLL1*2/BRAMBLING/5/BABAX/LR42//BABAX*2/4/SNI/TRAP#1/3/KAUZ*2/TRAP//KAUZ |
| 313 | 6177017 | WBLL1*2/BRAMBLING/5/BABAX/LR42//BABAX*2/4/SNI/TRAP#1/3/KAUZ*2/TRAP//KAUZ |
| 314 | 6177020 | WBLL1*2/BRAMBLING/5/BABAX/LR42//BABAX*2/4/SNI/TRAP#1/3/KAUZ*2/TRAP//KAUZ |
| 315 | 6177024 | WBLL1*2/BRAMBLING/5/BABAX/LR42//BABAX*2/4/SNI/TRAP#1/3/KAUZ*2/TRAP//KAUZ |
| 316 | 6177026 | WBLL1*2/BRAMBLING/5/BABAX/LR42//BABAX*2/4/SNI/TRAP#1/3/KAUZ*2/TRAP//KAUZ |
| 317 | 6177049 | PANDORA/3/KIRITATI//PRL/2*PASTOR |
| 318 | 6177051 | PANDORA/3/KIRITATI//PRL/2*PASTOR |
| 319 | 6177056 | PANDORA//WBLL1*2/BRAMBLING |
| 320 | 6177057 | PANDORA//WBLL1*2/BRAMBLING |
| 321 | 6177058 | PANDORA//WBLL1*2/BRAMBLING |
| 322 | 6177060 | ATTILA*2/PBW65//AKURI |
| 323 | 6177062 | ATTILA*2/PBW65//AKURI |
| 324 | 6177080 | ATTILA*2/PBW65//KBIRD |
| 325 | 6177095 | FRANCOLIN #1/HAWFINCH #1 |
| 326 | 6177099 | FRANCOLIN #1/KIRITATI |
| 327 | 6177100 | FRANCOLIN #1/KIRITATI |
| 328 | 6177126 | FRNCLN/TECUE #1 |
| 329 | 6177127 | FRNCLN/TECUE #1 |
| 330 | 6177139 | TRCH/HUIRIVIS #1 |
| 331 | 6177145 | TRCH/HUIRIVIS #1 |

| | | |
|---|---|---|
| 332 | 6177147 | TRCH/HUIRIVIS #1 |
| 333 | 6177148 | TRCH/HUIRIVIS #1 |
| 334 | 6177157 | TRCH/KBIRD |
| 335 | 6177158 | TRCH/KBIRD |
| 336 | 6177159 | TRCH/KBIRD |
| 337 | 6177174 | PFAU/SERI.1B//AMAD/3/WAXWING/4/HUIRIVIS #1 |
| 338 | 6177175 | PFAU/SERI.1B//AMAD/3/WAXWING/4/MUU |
| 339 | 6177189 | CHIBIA//PRLII/CM65531/3/FISCAL/4/CROSBILL #1 |
| 340 | 6177195 | VORB/FISCAL//AKURI #1 |
| 341 | 6177222 | MILAN/S87230//BAV92/3/JUCHI |
| 342 | 6177233 | MILAN/S87230//BAV92/3/AKURI #1 |
| 343 | 6177257 | PRL/2*PASTOR//CROSBILL #1 |
| 344 | 6177326 | ROLF07/MUU |
| 345 | 6177327 | ROLF07/MUU |
| 346 | 6177342 | ROLF07/KINGBIRD #1 |
| 347 | 6177369 | WBLL1*2/BRAMBLING//HUIRIVIS #1 |
| 348 | 6177393 | BECARD/AKURI |
| 349 | 6177394 | BECARD/AKURI |
| 350 | 6177395 | BECARD/AKURI |
| 351 | 6177396 | BECARD/AKURI |
| 352 | 6177402 | WBLL1*2/KKTS//KINGBIRD #1 |
| 353 | 6177403 | WBLL1*2/KKTS//KINGBIRD #1 |
| 354 | 6177404 | WBLL1*2/KKTS//KINGBIRD #1 |
| 355 | 6177405 | WBLL1*2/KKTS//KINGBIRD #1 |
| 356 | 6177406 | WBLL1*2/KKTS//KINGBIRD #1 |
| 357 | 6177407 | WBLL1*2/KKTS//KINGBIRD #1 |
| 358 | 6177408 | WBLL1*2/KKTS//KINGBIRD #1 |
| 359 | 6177411 | WBLL1*2/KKTS//KINGBIRD #1 |
| 360 | 6177412 | WBLL1*2/KKTS//KINGBIRD #1 |
| 361 | 6177413 | WBLL1*2/KKTS//KINGBIRD #1 |
| 362 | 6177439 | KBIRD//WBLL1*2/KURUKU |
| 363 | 6177447 | KBIRD//WBLL1*2/KURUKU |
| 364 | 6177452 | WBLL1/KUKUNA//TACUPETO F2001/6/PVN//CAR422/ANA/5/BOW/CROW//BUC/PVN/3/YR/4/TRAP#1 |
| 365 | 6177453 | WBLL1/KUKUNA//TACUPETO F2001/6/PVN//CAR422/ANA/5/BOW/CROW//BUC/PVN/3/YR/4/TRAP#1 |
| 366 | 6177498 | KACHU/MUU |
| 367 | 6177500 | KACHU/MUU |
| 368 | 6177509 | KINGBIRD #1/KACHU |
| 369 | 6177515 | KINGBIRD #1/KACHU |
| 370 | 6177544 | MILAN/S87230//BAV92/3/AKURI |
| 371 | 6177551 | TOBA97/PASTOR//KINGBIRD #1 |
| 372 | 6177552 | WAXWING/4/SNI/TRAP#1/3/KAUZ*2/TRAP//KAUZ/5/AKURI |
| 373 | 6177553 | WAXWING/4/SNI/TRAP#1/3/KAUZ*2/TRAP//KAUZ/5/AKURI |
| 374 | 6177554 | WAXWING/4/SNI/TRAP#1/3/KAUZ*2/TRAP//KAUZ/5/AKURI |

| | | |
|---|---|---|
| 375 | 6177555 | WAXWING/4/SNI/TRAP#1/3/KAUZ*2/TRAP//KAUZ/5/AKURI |
| 376 | 6177558 | WAXWING/4/SNI/TRAP#1/3/KAUZ*2/TRAP//KAUZ/5/AKURI |
| 377 | 6177560 | WAXWING/4/SNI/TRAP#1/3/KAUZ*2/TRAP//KAUZ/5/AKURI |
| 378 | 6177561 | WAXWING/4/SNI/TRAP#1/3/KAUZ*2/TRAP//KAUZ/5/TECUE #1 |
| 379 | 6177562 | WAXWING/4/SNI/TRAP#1/3/KAUZ*2/TRAP//KAUZ/5/TECUE #1 |
| 380 | 6177563 | WAXWING/4/SNI/TRAP#1/3/KAUZ*2/TRAP//KAUZ/5/TECUE #1 |
| 381 | 6177570 | WAXWING/4/SNI/TRAP#1/3/KAUZ*2/TRAP//KAUZ/5/TECUE #1 |
| 382 | 6177575 | WAXWING/4/SNI/TRAP#1/3/KAUZ*2/TRAP//KAUZ/5/KBIRD |
| 383 | 6177576 | WAXWING/4/SNI/TRAP#1/3/KAUZ*2/TRAP//KAUZ/5/KBIRD |
| 384 | 6177578 | WAXWING/4/SNI/TRAP#1/3/KAUZ*2/TRAP//KAUZ/5/KBIRD |
| 385 | 6177589 | KBIRD//INQALAB 91*2/KUKUNA |
| 386 | 6177595 | KINGBIRD #1//INQALAB 91*2/TUKURU |
| 387 | 6177598 | KINGBIRD #1//INQALAB 91*2/TUKURU |
| 388 | 6177599 | KINGBIRD #1//INQALAB 91*2/TUKURU |
| 389 | 6177600 | KINGBIRD #1//INQALAB 91*2/TUKURU |
| 390 | 6177603 | KINGBIRD #1//INQALAB 91*2/TUKURU |
| 391 | 6177627 | PBW343*2/KHVAKI//JUCHI |
| 392 | 6177636 | PBW343*2/KUKUNA//AKURI |
| 393 | 6177647 | PBW343*2/KUKUNA//TECUE #1 |
| 394 | 6177648 | PBW343*2/KUKUNA//TECUE #1 |
| 395 | 6177650 | PBW343*2/KUKUNA//TECUE #1 |
| 396 | 6177652 | PBW343*2/KUKUNA//TECUE #1 |
| 397 | 6177657 | PBW343*2/KUKUNA//TECUE #1 |
| 398 | 6177659 | PBW343*2/KUKUNA//TECUE #1 |
| 399 | 6177665 | PBW343*2/KUKUNA//TECUE #1 |
| 400 | 6177666 | PBW343*2/KUKUNA//TECUE #1 |
| 401 | 6177667 | PBW343*2/KUKUNA//TECUE #1 |
| 402 | 6177671 | PBW343*2/KUKUNA//TECUE #1 |
| 403 | 6177674 | JUCHI/HUIRIVIS #1 |
| 404 | 6177675 | JUCHI/HUIRIVIS #1 |
| 405 | 6177721 | HUIRIVIS #1/KBIRD |
| 406 | 6177732 | PVN//CAR422/ANA/5/BOW/CROW//BUC/PVN/3/YR/4/TRAP#1/6/AKURI #1 |
| 407 | 6177733 | PVN//CAR422/ANA/5/BOW/CROW//BUC/PVN/3/YR/4/TRAP#1/6/AKURI #1 |
| 408 | 6177735 | PVN//CAR422/ANA/5/BOW/CROW//BUC/PVN/3/YR/4/TRAP#1/6/AKURI #1 |
| 409 | 6177737 | FRANCOLIN #1/YANAC |
| 410 | 6177762 | BL2064//SW89-5124*2/FASAN/3/TILHI/5/KAUZ//ALTAR 84/AOS/3/KAUZ/4/SW94.15464 |
| 411 | 6177765 | BL2064//SW89-5124*2/FASAN/3/TILHI/5/KAUZ//ALTAR 84/AOS/3/KAUZ/4/SW94.15464 |
| 412 | 6177771 | BABAX/LR42//BABAX*2/3/KUKUNA/4/TAM200/PASTOR//TOBA97 |
| 413 | 6177772 | BABAX/LR42//BABAX*2/3/KUKUNA/4/TAM200/PASTOR//TOBA97 |
| 414 | 6177773 | BABAX/LR42//BABAX*2/3/KUKUNA/4/TINKIO #1 |
| 415 | 6177790 | QUAIU/YANAC |
| 416 | 6177824 | TAM200/PASTOR//TOBA97/3/WHEAR |
| 417 | 6177828 | FRNCLN/ROLF07 |

| | | |
|---|---|---|
| 418 | 6177829 | FRNCLN/ROLF07 |
| 419 | 6177830 | FRNCLN/ROLF07 |
| 420 | 6177834 | FRNCLN/ROLF07 |
| 421 | 6177837 | FRNCLN/BECARD |
| 422 | 6177838 | FRNCLN/BECARD |
| 423 | 6177841 | FRNCLN/BECARD |
| 424 | 6177844 | FRNCLN/BECARD |
| 425 | 6177845 | FRNCLN/BECARD |
| 426 | 6177846 | FRNCLN/BECARD |
| 427 | 6177851 | PARUS/FRANCOLIN #1 |
| 428 | 6177854 | PARUS/FRANCOLIN #1 |
| 429 | 6177858 | PARUS/FRANCOLIN #1 |
| 430 | 6177860 | PARUS/FRANCOLIN #1 |
| 431 | 6177861 | PARUS/FRANCOLIN #1 |
| 432 | 6177862 | PARUS/FRANCOLIN #1 |
| 433 | 6177869 | OASIS/SKAUZ//4*BCN/3/PASTOR/4/KAUZ*2/YACO//KAUZ/5/FRET2/TUKURU//FRET2/6/FRET2/TUKURU//FRET2 |
| 434 | 6177873 | OASIS/SKAUZ//4*BCN/3/PASTOR/4/KAUZ*2/YACO//KAUZ*2/5/KRONSTAD F2004 |
| 435 | 6177898 | ATTILA*2/PBW65//MUU #1/3/FRANCOLIN #1 |
| 436 | 6177900 | ATTILA*2/PBW65//MUU #1/3/FRANCOLIN #1 |
| 437 | 6177920 | C80.1/3*BATAVIA//2*WBLL1/3/WBLL1*2/TUKURU/4/FRET2/TUKURU//FRET2 |
| 438 | 6177929 | SAUAL/3/C80.1/3*BATAVIA//2*WBLL1/4/SITE/MO//PASTOR/3/TILHI |
| 439 | 6177930 | MURGA/3/GAN/AE.SQUARROSA (408)//2*OASIS/5*BORL95/4/FRANCOLIN #1 |
| 440 | 6177946 | UP2338*2/VIVITSI/3/FRET2/TUKURU//FRET2/4/MISR 1 |
| 441 | 6177947 | UP2338*2/VIVITSI/3/FRET2/TUKURU//FRET2/4/MISR 1 |
| 442 | 6177950 | UP2338*2/VIVITSI/3/FRET2/TUKURU//FRET2/4/MISR 1 |
| 443 | 6177951 | UP2338*2/VIVITSI/3/FRET2/TUKURU//FRET2/4/MISR 1 |
| 444 | 6177962 | HUIRIVIS #1*2/MURGA |
| 445 | 6177968 | HUIRIVIS #1*2/MURGA |
| 446 | 6177980 | CONI#1/2*HUIRIVIS #1 |
| 447 | 6178005 | TECUE #1/2*WAXWING |
| 448 | 6178012 | MUU/4/BAV92//IRENA/KAUZ/3/HUITES/5/BAV92//IRENA/KAUZ/3/HUITES |
| 449 | 6178013 | KIRITATI/4/2*BAV92//IRENA/KAUZ/3/HUITES |
| 450 | 6178017 | KIRITATI/4/2*BAV92//IRENA/KAUZ/3/HUITES |
| 451 | 6178018 | KIRITATI/4/2*BAV92//IRENA/KAUZ/3/HUITES |
| 452 | 6178019 | KIRITATI/4/2*BAV92//IRENA/KAUZ/3/HUITES |
| 453 | 6178020 | KIRITATI/4/2*BAV92//IRENA/KAUZ/3/HUITES |
| 454 | 6178021 | KIRITATI/4/2*BAV92//IRENA/KAUZ/3/HUITES |
| 455 | 6178029 | PFAU/WEAVER//KIRITATI/3/FRET2/TUKURU//FRET2/4/FRET2/TUKURU//FRET2 |
| 456 | 6178040 | MUU/5/WBLL1*2/4/YACO/PBW65/3/KAUZ*2/TRAP//KAUZ/6/WBLL1*2/4/SNI/TRAP#1/3/KAUZ*2/TRAP//KAUZ |
| 457 | 6178056 | SKAUZ/BAV92//2*WBLL1*2/KKTS |
| 458 | 6178080 | KBIRD//WH 542/2*PASTOR/3/WBLL1*2/BRAMBLING |
| 459 | 6178095 | PVN/5/2*REH/HARE//2*BCN/3/CROC_1/AE.SQUARROSA (213)//PGO/4/HUITES |

| | | |
|---|---|---|
| 460 | 6178118 | KZA/5/2*WBLL1/3/STAR//KAUZ/STAR/4/BAV92/RAYON |
| 461 | 6178123 | KZA//WH 542/2*PASTOR/3/BACEU #1 |
| 462 | 6178134 | KSW/SAUAL//SAUAL |
| 463 | 6178136 | KSW/SAUAL//SAUAL |
| 464 | 6178166 | KACHU/6/NG8201/KAUZ/4/SHA7//PRL/VEE#6/3/FASAN/5/MILAN/KAUZ/7/KACHU |
| 465 | 6178176 | KACHU/6/NG8201/KAUZ/4/SHA7//PRL/VEE#6/3/FASAN/5/MILAN/KAUZ/7/KACHU |
| 466 | 6178184 | KACHU/6/NG8201/KAUZ/4/SHA7//PRL/VEE#6/3/FASAN/5/MILAN/KAUZ/7/KACHU |
| 467 | 6178193 | TUKURU//BAV92/RAYON*2/3/JUCHI |
| 468 | 6178199 | TUKURU//BAV92/RAYON*2/3/PVN |
| 469 | 6178200 | TUKURU//BAV92/RAYON*2/3/PVN |
| 470 | 6178201 | TUKURU//BAV92/RAYON*2/3/PVN |
| 471 | 6178205 | TUKURU//BAV92/RAYON*2/3/PVN |
| 472 | 6178206 | TUKURU//BAV92/RAYON*2/3/PVN |
| 473 | 6178225 | PBW343/PASTOR*2//K6295.4A |
| 474 | 6178238 | UP2338*2/KKTS*2//YANAC |
| 475 | 6178240 | UP2338*2/KKTS*2//YANAC |
| 476 | 6178241 | UP2338*2/KKTS*2//YANAC |
| 477 | 6178243 | UP2338*2/KKTS*2//YANAC |
| 478 | 6178244 | UP2338*2/KKTS*2//YANAC |
| 479 | 6178246 | TILILA/JUCHI/4/SERI.1B//KAUZ/HEVO/3/AMAD |
| 480 | 6178247 | TILILA/JUCHI/4/SERI.1B//KAUZ/HEVO/3/AMAD |
| 481 | 6178248 | TILILA/JUCHI/4/SERI.1B//KAUZ/HEVO/3/AMAD |
| 482 | 6178273 | WAXWING*2/DIAMONDBIRD |
| 483 | 6178296 | WAXWING/KIRITATI*2//YANAC |
| 484 | 6178298 | WAXWING/KIRITATI*2//YANAC |
| 485 | 6178331 | BAV92//IRENA/KAUZ/3/HUITES*2/4/DIAMONDBIRD |
| 486 | 6178335 | BAV92//IRENA/KAUZ/3/HUITES*2/4/MILAN/KAUZ//CHIL/CHUM18 |
| 487 | 6178362 | BAV92//IRENA/KAUZ/3/HUITES*2/4/PVN |
| 488 | 6178366 | CNO79//PF70354/MUS/3/PASTOR/4/BAV92*2/5/HAR311 |
| 489 | 6178368 | CNO79//PF70354/MUS/3/PASTOR/4/BAV92*2/5/HAR311 |
| 490 | 6178369 | CNO79//PF70354/MUS/3/PASTOR/4/BAV92*2/5/HAR311 |
| 491 | 6178370 | CNO79//PF70354/MUS/3/PASTOR/4/BAV92*2/5/HAR311 |
| 492 | 6178372 | CNO79//PF70354/MUS/3/PASTOR/4/BAV92*2/5/HAR311 |
| 493 | 6178376 | CNO79//PF70354/MUS/3/PASTOR/4/BAV92*2/5/HAR311 |
| 494 | 6178378 | CNO79//PF70354/MUS/3/PASTOR/4/BAV92*2/5/HAR311 |
| 495 | 6178379 | CNO79//PF70354/MUS/3/PASTOR/4/BAV92*2/5/HAR311 |
| 496 | 6178383 | CNO79//PF70354/MUS/3/PASTOR/4/BAV92*2/5/HAR311 |
| 497 | 6178386 | CNO79//PF70354/MUS/3/PASTOR/4/BAV92*2/5/HAR311 |
| 498 | 6178391 | CNO79//PF70354/MUS/3/PASTOR/4/BAV92*2/5/HAR311 |
| 499 | 6178393 | CNO79//PF70354/MUS/3/PASTOR/4/BAV92*2/5/HAR311 |
| 500 | 6178394 | CNO79//PF70354/MUS/3/PASTOR/4/BAV92*2/5/HAR311 |
| 501 | 6178397 | CNO79//PF70354/MUS/3/PASTOR/4/BAV92*2/5/FH6-1-7 |
| 502 | 6178398 | CNO79//PF70354/MUS/3/PASTOR/4/BAV92*2/5/FH6-1-7 |

| | | |
|---|---|---|
| 503 | 6178401 | CNO79//PF70354/MUS/3/PASTOR/4/BAV92*2/5/FH6-1-7 |
| 504 | 6178402 | CNO79//PF70354/MUS/3/PASTOR/4/BAV92*2/5/FH6-1-7 |
| 505 | 6178403 | CNO79//PF70354/MUS/3/PASTOR/4/BAV92*2/5/FH6-1-7 |
| 506 | 6178404 | CNO79//PF70354/MUS/3/PASTOR/4/BAV92*2/5/FH6-1-7 |
| 507 | 6178405 | CNO79//PF70354/MUS/3/PASTOR/4/BAV92*2/5/FH6-1-7 |
| 508 | 6178406 | CNO79//PF70354/MUS/3/PASTOR/4/BAV92*2/5/FH6-1-7 |
| 509 | 6178423 | ROLF07*2/YANAC |
| 510 | 6178424 | ROLF07*2/YANAC |
| 511 | 6178426 | ROLF07*2/DIAMONDBIRD |
| 512 | 6178429 | ROLF07*2/DIAMONDBIRD |
| 513 | 6178431 | ROLF07*2/DIAMONDBIRD |
| 514 | 6178459 | WBLL1/KUKUNA//TACUPETO F2001*2/3/HAR311 |
| 515 | 6178476 | WBLL1/DIAMONDBIRD//WBLL1*2/VIVITSI |
| 516 | 6178477 | WBLL1/DIAMONDBIRD//WBLL1*2/VIVITSI |
| 517 | 6178484 | WBLL1*2/VIVITSI*2/6/PVN//CAR422/ANA/5/BOW/CROW//BUC/PVN/3/YR/4/TRAP#1 |
| 518 | 6178485 | WBLL1*2/VIVITSI*2/6/PVN//CAR422/ANA/5/BOW/CROW//BUC/PVN/3/YR/4/TRAP#1 |
| 519 | 6178517 | KACHU/KIRITATI//KACHU |
| 520 | 6178526 | SAUAL/YANAC//SAUAL |
| 521 | 6178527 | SAUAL/YANAC//SAUAL |
| 522 | 6178528 | SAUAL/YANAC//SAUAL |
| 523 | 6178529 | SAUAL/YANAC//SAUAL |
| 524 | 6178530 | SAUAL/YANAC//SAUAL |
| 525 | 6178531 | SAUAL/YANAC//SAUAL |
| 526 | 6178533 | SAUAL/YANAC//SAUAL |
| 527 | 6178534 | SAUAL/YANAC//SAUAL |
| 528 | 6178537 | SAUAL/KIRITATI//SAUAL |
| 529 | 6178538 | SAUAL/KIRITATI//SAUAL |
| 530 | 6178539 | SAUAL/KIRITATI//SAUAL |
| 531 | 6178541 | SAUAL/KIRITATI//SAUAL |
| 532 | 6178556 | PRL/2*PASTOR*2//FH6-1-7 |
| 533 | 6178557 | PRL/2*PASTOR*2//FH6-1-7 |
| 534 | 6178564 | PRL/2*PASTOR*2//K6295.4A |
| 535 | 6178575 | CS/TH.SC//3*PVN/3/MIRLO/BUC/4/URES/JUN//KAUZ/5/HUITES/6/YANAC/7/CS/TH.SC//3*PVN/3/MIRLO/BUC/4/MILAN/5/TILHI |
| 536 | 6178591 | FINSI/METSO//FH6-1-7/3/FINSI/METSO |
| 537 | 6178600 | FINSI/METSO//FH6-1-7/3/FINSI/METSO |
| 538 | 6178633 | PF74354//LD/ALD/4/2*BR12*2/3/JUP//PAR214*6/FB6631/5/NL750/6/PVN/7/TOBA97/PASTOR |
| 539 | 6178654 | PBW343*2/KHVAKI*2/6/PVN//CAR422/ANA/5/BOW/CROW//BUC/PVN/3/YR/4/TRAP#1 |
| 540 | 6178681 | C80.1/3*BATAVIA//2*WBLL1/4/CROC_1/AE.SQUARROSA (224)//KULIN/3/WESTONIA/5/WBLL1*2/KURUKU |
| 541 | 6178701 | C80.1/3*BATAVIA//2*WBLL1*2/3/WBLL1*2/TUKURU |
| 542 | 6178714 | WAXWING/KIRITATI*2/3/C80.1/3*BATAVIA//2*WBLL1 |
| 543 | 6178715 | WAXWING/KIRITATI*2/3/C80.1/3*BATAVIA//2*WBLL1 |
| 544 | 6178716 | WAXWING/KIRITATI*2/3/C80.1/3*BATAVIA//2*WBLL1 |

| | | |
|---|---|---|
| 545 | 6178717 | WAXWING/KIRITATI*2/3/C80.1/3*BATAVIA//2*WBLL1 |
| 546 | 6178726 | BAV92//IRENA/KAUZ/3/HUITES*2/4/WHEAR |
| 547 | 6178728 | BAV92//IRENA/KAUZ/3/HUITES*2/4/WHEAR |
| 548 | 6178734 | BAV92//IRENA/KAUZ/3/HUITES*2/4/WHEAR |
| 549 | 6178737 | BAV92//IRENA/KAUZ/3/HUITES*2/4/WHEAR |
| 550 | 6178739 | BAV92//IRENA/KAUZ/3/HUITES*2/4/WHEAR |
| 551 | 6178752 | FRET2/KIRITATI/3/C80.1/3*BATAVIA//2*WBLL1/4/FRET2/TUKURU//FRET2 |
| 552 | 6178759 | KACHU*2/WHEAR |
| 553 | 6178760 | KACHU*2/WHEAR |
| 554 | 6178763 | KACHU*2/WHEAR |
| 555 | 6178768 | KACHU/3/C80.1/3*BATAVIA//2*WBLL1/4/KACHU |
| 556 | 6178770 | KACHU/3/C80.1/3*BATAVIA//2*WBLL1/4/KACHU |
| 557 | 6178775 | KACHU/3/C80.1/3*BATAVIA//2*WBLL1/4/KACHU |
| 558 | 6178783 | SAUAL/WHEAR//SAUAL |
| 559 | 6178788 | SAUAL/WHEAR//SAUAL |
| 560 | 6178789 | SAUAL/WHEAR//SAUAL |
| 561 | 6178790 | SAUAL/WHEAR//SAUAL |
| 562 | 6178797 | SAUAL/3/C80.1/3*BATAVIA//2*WBLL1/4/SAUAL |
| 563 | 6178819 | SAUAL/3/SW89.3064//CMH82.17/SERI/4/SAUAL |
| 564 | 6178820 | SAUAL/3/SW89.3064//CMH82.17/SERI/4/SAUAL |
| 565 | 6178822 | SAUAL/3/SW89.3064//CMH82.17/SERI/4/SAUAL |
| 566 | 6178824 | SAUAL/3/SW89.3064//CMH82.17/SERI/4/SAUAL |
| 567 | 6178828 | SAUAL*2/3/WL6718//2*PRL/VEE#6 |
| 568 | 6178833 | SAUAL*2/3/WL6718//2*PRL/VEE#6 |
| 569 | 6178834 | SAUAL*2/3/WL6718//2*PRL/VEE#6 |
| 570 | 6178836 | SAUAL*2/3/WL6718//2*PRL/VEE#6 |
| 571 | 6178837 | SAUAL*2/3/WL6718//2*PRL/VEE#6 |
| 572 | 6178838 | SAUAL*2/3/WL6718//2*PRL/VEE#6 |
| 573 | 6178841 | SAUAL*2/3/WL6718//2*PRL/VEE#6 |
| 574 | 6178846 | SAUAL*2/3/WL6718//2*PRL/VEE#6 |
| 575 | 6178847 | SAUAL*2/3/WL6718//2*PRL/VEE#6 |
| 576 | 6178875 | ROLF07*2/5/FCT/3/GOV/AZ//MUS/4/DOVE/BUC |
| 577 | 6178881 | WBLL1/4/BOW/NKT//CBRD/3/CBRD/5/WBLL1*2/TUKURU |
| 578 | 6178888 | WBLL1/4/BOW/NKT//CBRD/3/CBRD/5/WBLL1*2/TUKURU |
| 579 | 6178896 | KACHU*2//CHIL/CHUM18 |
| 580 | 6178897 | KACHU*2//CHIL/CHUM18 |
| 581 | 6178898 | KACHU*2//CHIL/CHUM18 |
| 582 | 6178900 | KACHU*2//CHIL/CHUM18 |
| 583 | 6178901 | KACHU*2//CHIL/CHUM18 |
| 584 | 6178902 | KACHU*2//CHIL/CHUM18 |
| 585 | 6178907 | SAUAL/3/SHA3/SERI//SHA4/LIRA/4/SAUAL |
| 586 | 6178908 | SAUAL/3/SHA3/SERI//SHA4/LIRA/4/SAUAL |
| 587 | 6178918 | PBW343/PASTOR*2/6/TURACO/5/CHIR3/4/SIREN//ALTAR 84/AE.SQUARROSA (205)/3/3*BUC |

| | | |
|---|---|---|
| 588 | 6178935 | WBLL1*2/TUKURU//FN/2*PASTOR/3/FRET2/KIRITATI |
| 589 | 6178943 | WAXWING/SRTU//WAXWING/KIRITATI |
| 590 | 6178949 | ATTILA*2/PBW65*2//TNMU |
| 591 | 6178950 | ATTILA*2/PBW65*2//TNMU |
| 592 | 6178964 | FRET2*2/4/SNI/TRAP#1/3/KAUZ*2/TRAP//KAUZ*2/5/BOW/URES//2*WEAVER/3/CROC_1/AE.SQUARROSA (213)//PGO |
| 593 | 6178965 | FRET2*2/4/SNI/TRAP#1/3/KAUZ*2/TRAP//KAUZ*2/5/BOW/URES//2*WEAVER/3/CROC_1/AE.SQUARROSA (213)//PGO |
| 594 | 6178971 | SAAR/2*WAXWING |
| 595 | 6178972 | PFAU/SERI.1B//AMAD/3/WAXWING/4/BABAX/LR42//BABAX*2/3/KURUKU |
| 596 | 6178973 | PFAU/SERI.1B//AMAD/3/WAXWING/4/BABAX/LR42//BABAX*2/3/KURUKU |
| 597 | 6178974 | PFAU/SERI.1B//AMAD/3/WAXWING/4/BABAX/LR42//BABAX*2/3/KURUKU |
| 598 | 6178981 | PFAU/SERI.1B//AMAD/3/WAXWING/4/BABAX/LR42//BABAX*2/3/KURUKU |
| 599 | 6178996 | QUAIU/5/FRET2*2/4/SNI/TRAP#1/3/KAUZ*2/TRAP//KAUZ |
| 600 | 6178997 | QUAIU/5/FRET2*2/4/SNI/TRAP#1/3/KAUZ*2/TRAP//KAUZ |
| 601 | 6178999 | QUAIU/5/FRET2*2/4/SNI/TRAP#1/3/KAUZ*2/TRAP//KAUZ |
| 602 | 6179000 | QUAIU/5/FRET2*2/4/SNI/TRAP#1/3/KAUZ*2/TRAP//KAUZ |
| 603 | 6179010 | BABAX/LR42//BABAX*2/4/SNI/TRAP#1/3/KAUZ*2/TRAP//KAUZ/5/WBLL1*2/TUKURU |
| 604 | 6179012 | WBLL1*2/BRAMBLING/4/BABAX/LR42//BABAX*2/3/KURUKU |
| 605 | 6179013 | WBLL1*2/BRAMBLING/4/BABAX/LR42//BABAX*2/3/KURUKU |
| 606 | 6179017 | PFAU/WEAVER*2//BRAMBLING/3/QUAIU |
| 607 | 6179044 | QUAIU/TECUE #1 |
| 608 | 6179047 | QUAIU/TECUE #1 |
| 609 | 6179064 | WBLL1*2/CHAPIO//HAWFINCH #1 |
| 610 | 6179077 | WBLL1*2/KUKUNA//AKURI #1 |
| 611 | 6179078 | WBLL1*2/TUKURU//CROSBILL #1 |
| 612 | 6179079 | WBLL1*2/TUKURU//CROSBILL #1 |
| 613 | 6179082 | WBLL1*2/TUKURU//CROSBILL #1 |
| 614 | 6179087 | REH/HARE//2*BCN/3/CROC_1/AE.SQUARROSA (213)//PGO/4/HUITES/5/PVN |
| 615 | 6179088 | REH/HARE//2*BCN/3/CROC_1/AE.SQUARROSA (213)//PGO/4/HUITES/5/PVN |
| 616 | 6179095 | PFAU/WEAVER*2//BRAMBLING/3/AKURI #1 |
| 617 | 6179116 | PBW343*2/KHVAKI/5/KAUZ//ALTAR 84/AOS/3/KAUZ/4/SW94.15464 |
| 618 | 6179118 | PAURAQ/4/PFAU/SERI.1B//AMAD/3/WAXWING |
| 619 | 6179122 | C80.1/3*BATAVIA//2*WBLL1/3/2*FRET2/TUKURU//FRET2 |
| 620 | 6179125 | C80.1/3*BATAVIA//2*WBLL1/3/2*FRET2/TUKURU//FRET2 |
| 621 | 6179128 | TACUPETO F2001/SAUAL/4/BABAX/LR42//BABAX*2/3/KURUKU |
| 622 | 6179129 | TACUPETO F2001/SAUAL/4/BABAX/LR42//BABAX*2/3/KURUKU |
| 623 | 6179130 | TACUPETO F2001/SAUAL/4/BABAX/LR42//BABAX*2/3/KURUKU |
| 624 | 6179132 | TACUPETO F2001/SAUAL/4/BABAX/LR42//BABAX*2/3/KURUKU |
| 625 | 6179136 | TACUPETO F2001/SAUAL/4/BABAX/LR42//BABAX*2/3/KURUKU |
| 626 | 6179141 | CHEN/AE.SQ//WEAVER/3/SSERI1/4/TOBA97/PASTOR/5/MUU #1 |
| 627 | 6179143 | CHEN/AE.SQ//WEAVER/3/SSERI1/4/TOBA97/PASTOR/5/MUU #1 |
| 628 | 6179148 | MUU #1/SAUAL//MUU |
| 629 | 6179151 | MUU #1//PBW343*2/KUKUNA/3/MUU |

| | | |
|---|---|---|
| 630 | 6179152 | MUU #1//PBW343*2/KUKUNA/3/MUU |
| 631 | 6179156 | MUU #1//PBW343*2/KUKUNA/3/MUU |
| 632 | 6179159 | MUU #1//PBW343*2/KUKUNA/3/MUU |
| 633 | 6179165 | BABAX/LR42//BABAX/3/BABAX/LR42//BABAX/4/ATTILA/2*PASTOR/5/QUAIU #3 |
| 634 | 6179184 | OASIS/SKAUZ//4*BCN/3/2*PASTOR/5/FRET2*2/4/SNI/TRAP#1/3/KAUZ*2/TRAP//KAUZ/6/SAUAL |
| 635 | 6179185 | OASIS/SKAUZ//4*BCN/3/2*PASTOR/5/FRET2*2/4/SNI/TRAP#1/3/KAUZ*2/TRAP//KAUZ/6/SAUAL |
| 636 | 6179207 | KFA/2*KACHU |
| 637 | 6179216 | KFA/2*KACHU |
| 638 | 6179218 | KFA/2*KACHU |
| 639 | 6179220 | KFA/2*KACHU |
| 640 | 6179221 | KFA/2*KACHU |
| 641 | 6179222 | PBW343*2/KUKUNA*2//FRTL/PIFED |
| 642 | 6179223 | PBW343*2/KUKUNA*2//FRTL/PIFED |
| 643 | 6179225 | PBW343*2/KUKUNA*2//FRTL/PIFED |
| 644 | 6179226 | PBW343*2/KUKUNA*2//FRTL/PIFED |
| 645 | 6179227 | PBW343*2/KUKUNA*2//FRTL/PIFED |
| 646 | 6179228 | PBW343*2/KUKUNA*2//FRTL/PIFED |
| 647 | 6179236 | INQALAB 91*2/KUKUNA*2//PVN |
| 648 | 6179244 | INQALAB 91*2/KUKUNA*2//PVN |
| 649 | 6179247 | KANCHAN*2/JUCHI |
| 650 | 6179249 | KANCHAN*2/JUCHI |
| 651 | 6179251 | TILILA/TUKURU/4/SERI.1B*2/3/KAUZ*2/BOW//KAUZ |
| 652 | 6179253 | WBLL1*2/4/BABAX/LR42//BABAX/3/BABAX/LR42//BABAX |
| 653 | 6179254 | WBLL1*2/4/BABAX/LR42//BABAX/3/BABAX/LR42//BABAX |
| 654 | 6179255 | WBLL1*2/4/BABAX/LR42//BABAX/3/BABAX/LR42//BABAX |
| 655 | 6179261 | BABAX/LR42//BABAX/3/BABAX/LR42//BABAX/4/DIAMONDBIRD/5/FRET2/TUKURU//FRET2 |
| 656 | 6179271 | TACUPETO F2001/6/CNDO/R143//ENTE/MEXI_2/3/AEGILOPS SQUARROSA (TAUS)/4/WEAVER/5/PASTOR/7/ROLF07 |
| 657 | 6179272 | TACUPETO F2001/6/CNDO/R143//ENTE/MEXI_2/3/AEGILOPS SQUARROSA (TAUS)/4/WEAVER/5/PASTOR/7/ROLF07 |
| 658 | 6179273 | TACUPETO F2001/6/CNDO/R143//ENTE/MEXI_2/3/AEGILOPS SQUARROSA (TAUS)/4/WEAVER/5/PASTOR/7/ROLF07 |
| 659 | 6179274 | TACUPETO F2001/6/CNDO/R143//ENTE/MEXI_2/3/AEGILOPS SQUARROSA (TAUS)/4/WEAVER/5/PASTOR/7/ROLF07 |
| 660 | 6179275 | TACUPETO F2001/6/CNDO/R143//ENTE/MEXI_2/3/AEGILOPS SQUARROSA (TAUS)/4/WEAVER/5/PASTOR/7/ROLF07 |
| 661 | 6179276 | TACUPETO F2001/6/CNDO/R143//ENTE/MEXI_2/3/AEGILOPS SQUARROSA (TAUS)/4/WEAVER/5/PASTOR/7/ROLF07 |
| 662 | 6179277 | TACUPETO F2001/6/CNDO/R143//ENTE/MEXI_2/3/AEGILOPS SQUARROSA (TAUS)/4/WEAVER/5/PASTOR/7/ROLF07 |
| 663 | 6179286 | WBLL1*2/KUKUNA//KIRITATI/3/WBLL1*2/KUKUNA |
| 664 | 6179291 | WBLL1*2/KUKUNA//KIRITATI/3/WBLL1*2/KUKUNA |
| 665 | 6179293 | WBLL1*2/KUKUNA//KIRITATI/3/WBLL1*2/KUKUNA |
| 666 | 6179294 | WBLL1*2/KUKUNA//KIRITATI/3/WBLL1*2/KUKUNA |
| 667 | 6179296 | WBLL1*2/KUKUNA//KIRITATI/3/WBLL1*2/KUKUNA |
| 668 | 6179298 | WBLL1*2/KUKUNA//KIRITATI/3/WBLL1*2/KUKUNA |
| 669 | 6179318 | KAUZ/PASTOR//PBW343/3/HAR311/5/OASIS/SKAUZ//4*BCN/3/PASTOR/4/KAUZ*2/YACO//KAUZ |
| 670 | 6179322 | KAUZ/PASTOR//PBW343/3/HAR311/5/OASIS/SKAUZ//4*BCN/3/PASTOR/4/KAUZ*2/YACO//KAUZ |

| | | |
|---|---|---|
| 671 | 6179325 | C80.1/3*BATAVIA//2*WBLL1*2/3/PBW343/PASTOR |
| 672 | 6179332 | WBLL1/KUKUNA//TACUPETO F2001*2/3/C80.1/3*BATAVIA//2*WBLL1 |
| 673 | 6179344 | FRANCOLIN #1/4/BABAX/LR42//BABAX*2/3/KURUKU |
| 674 | 6179345 | FRANCOLIN #1/4/BABAX/LR42//BABAX*2/3/KURUKU |
| 675 | 6179346 | FRANCOLIN #1/4/BABAX/LR42//BABAX*2/3/KURUKU |
| 676 | 6179347 | FRANCOLIN #1/4/BABAX/LR42//BABAX*2/3/KURUKU |
| 677 | 6179348 | FRANCOLIN #1/4/BABAX/LR42//BABAX*2/3/KURUKU |
| 678 | 6179353 | PANDORA/4/BABAX/LR42//BABAX*2/3/KURUKU |
| 679 | 6179362 | KSW/5/2*ALTAR 84/AE.SQUARROSA (221)//3*BORL95/3/URES/JUN//KAUZ/4/WBLL1 |
| 680 | 6179370 | KSW/5/2*ALTAR 84/AE.SQUARROSA (221)//3*BORL95/3/URES/JUN//KAUZ/4/WBLL1 |
| 681 | 6179386 | OTUS/TOBA97//YANAC/3/TOBA97/ATTILA |
| 682 | 6179403 | WBLL1*2/4/BABAX/LR42//BABAX/3/BABAX/LR42//BABAX |
| 683 | 6179417 | CHIL/CHUM18//GONDO |
| 684 | 6179423 | KACHU/3/SHA3/SERI//SHA4/LIRA/4/KACHU |
| 685 | 6179457 | TACUPETO F2001//WBLL1*2/KKTS/3/WBLL1*2/BRAMBLING |
| 686 | 6179463 | WBLL1*2/KURUKU//KRONSTAD F2004/3/WBLL1*2/BRAMBLING |
| 687 | 6179464 | WBLL1*2/KURUKU//KRONSTAD F2004/3/WBLL1*2/BRAMBLING |
| 688 | 6179465 | WBLL1*2/TUKURU*2//KRONSTAD F2004 |
| 689 | 6179466 | WBLL1*2/TUKURU*2//KRONSTAD F2004 |
| 690 | 6179469 | WBLL1*2/VIVITSI//KRONSTAD F2004/3/WBLL1*2/BRAMBLING |
| 691 | 6179470 | WBLL1/4/HD2281/TRAP#1/3/KAUZ*2/TRAP//KAUZ/5/TACUPETO F2001/6/WBLL1*2/KKTS/7/WBLL1*2/BRAMBLING |
| 692 | 6179471 | WBLL1/KUKUNA//TACUPETO F2001/3/KRONSTAD F2004/4/ROLF07 |
| 693 | 6179473 | WBLL1/KUKUNA//TACUPETO F2001/3/KRONSTAD F2004/4/ROLF07 |
| 694 | 6179474 | WBLL1/KUKUNA//TACUPETO F2001/3/KRONSTAD F2004/4/ROLF07 |
| 695 | 6179475 | PRL/2*PASTOR*2//VORB |
| 696 | 6179479 | ATTILA*2/PBW65//KRONSTAD F2004 |
| 697 | 6179481 | KAUZ/PASTOR//PBW343/3/KRONSTAD F2004 |
| 698 | 6179497 | WBLL1*2/TUKURU//KRONSTAD F2004 |
| 699 | 6179508 | YAV_3/SCO//JO69/CRA/3/YAV79/4/AE.SQUARROSA (498)/5/LINE 1073/6/KAUZ*2/4/CAR//KAL/BB/3/NAC/5/KAUZ/7/KRONSTAD F2004 |
| 700 | 6179510 | PRL/2*PASTOR//VORB |
| 701 | 6179526 | BAV92//IRENA/KAUZ/3/HUITES*2/6/TURACO/5/CHIR3/4/SIREN//ALTAR 84/AE.SQUARROSA (205)/3/3*BUC |
| 702 | 6179527 | BAV92//IRENA/KAUZ/3/HUITES*2/6/TURACO/5/CHIR3/4/SIREN//ALTAR 84/AE.SQUARROSA (205)/3/3*BUC |
| 703 | 6179534 | TRCH*2/3/WUH1/VEE#5//CBRD |
| 704 | 6179538 | WBLL1*2/4/YACO/PBW65/3/KAUZ*2/TRAP//KAUZ*2/5/CHUANMAI 32 |
| 705 | 6179542 | WBLL1*2/KURUKU/4/BABAX/LR42//BABAX*2/3/KURUKU |
| 706 | 6179543 | WBLL1*2/KURUKU/4/BABAX/LR42//BABAX*2/3/KURUKU |
| 707 | 6179553 | MURGA/KRONSTAD F2004//QUAIU #3 |
| 708 | 6179559 | KFA/3/PFAU/WEAVER//BRAMBLING/4/PFAU/WEAVER*2//BRAMBLING |
| 709 | 6179562 | PBW343*2/KHVAKI*2//YANAC |
| 710 | 6179596 | BECARD/KACHU |
| 711 | 6179621 | FRET2/TUKURU//FRET2/3/KRONSTAD F2004/4/FRET2/TUKURU//FRET2 |
| 712 | 6179640 | MUNAL #1 |

| | | |
|---|---|---|
| 713 | 6181746 | WBLL1*2/BRAMBLING//JUCHI |
| 714 | 6181747 | BABAX/LR42//BABAX/3/BABAX/LR42//BABAX/4/ATTILA/2*PASTOR/5/QUAIU #3 |
| 715 | 6181748 | BABAX/LR42//BABAX/3/BABAX/LR42//BABAX/4/ATTILA/2*PASTOR/5/QUAIU #3 |
| 716 | 6181749 | BABAX/LR42//BABAX/3/BABAX/LR42//BABAX/4/ATTILA/2*PASTOR/5/QUAIU #3 |
| 717 | 6181750 | KENYA NYANGUMI/3/2*KAUZ/PASTOR//PBW343 |
| 718 | 6181755 | TILILA/JUCHI/4/SERI.1B//KAUZ/HEVO/3/AMAD |
| 719 | 6181759 | HEILO/7/IVAN/6/SABUF/5/BCN/4/RABI//GS/CRA/3/AE.SQUARROSA (190)/8/VORB/FISCAL |
| 720 | 6181760 | MUNAL #1 |

Supplementary Table S2 Trait adjusted means under three irrigation treatments (Bed 5IR, Flat 5IR and Bed ZT 5IR), two drought stress treatments (Bed drip and Bed 2IR) and one heat stress (Bed heat) treatment and their heritabilities across environments

| Trait | Environments | | | | | | | | | | | | | | | | | | |
|---|---|---|---|---|---|---|---|---|---|---|---|---|---|---|---|---|---|---|
| | Bed 5 IR (2009-2010) | | | Flat 5IR (2010-2011) | | | Bed ZT 5IR (2010-2011) | | | Bed drip (2010-2011) | | | Bed 2IR (2010-2011) | | | Bed Heat (2010-2011) | | | $h^2$ (combined environments) |
| | Min | Max | Mean | Min | Max | Mean | Min | Max | Mean | Min | Max | Mean | Min | Max | Mean | Min | Max | Mean | |
| GY | 5.87 | 9.19 | 7.70 | 4.82 | 9.22 | 7.64 | 4.53 | 8.34 | 6.62 | 0.01 | 2.91 | 1.26 | 1.51 | 5.15 | 3.59 | 1.90 | 5.15 | 3.73 | 0.54 |
| DH | 73 | 102 | 88.55 | 74 | 107 | 91.78 | 80 | 111 | 95.16 | 72 | 99 | 86.54 | 77 | 100 | 91.11 | 54 | 71 | 62.39 | 0.94 |
| PH | 87 | 120 | 104.8 | 95 | 127 | 113.0 | 77 | 121 | 99.82 | - | - | - | 56 | 98 | 77.65 | 55 | 95 | 76.40 | 0.76 |

GY Grain yield in tons per hectare

DH Days to heading

PH Plant height

Supplementary Table S3 Descriptive statistics of susceptibility indices and yield stability coefficient $P_i$

| Index | Min | Max | Average |
|---|---|---|---|
| DSI | 0.70 | 1.23 | 1.00 |
| HSI | 0.51 | 1.66 | 1.02 |
| Yld St ($P_i$) | 0.34 | 3.55 | 1.42 |

DSI Drought susceptibility index

HSI Heat susceptibility index

$P_i$ Superiority index

Supplementary Table S4 Pearson correlation coefficient (r) of grain yield (GY) with days to heading (DH) and plant height (PH) under multiple environments

| Traits | Bed 5IR (2009-2010) | Bed ZT 5IR (2010-2011) | Flat 5IR (2010-2011) | Bed drip (2010-2011) | Bed 2IR (2010-2011) | Bed heat (2010-2011) |
|---|---|---|---|---|---|---|
| GY and DH | 0.358<br>P<0.0001 | 0.158<br>P<0.05 | -0.347<br>P<0.0001 | -0.641<br>P<0.0001 | -0.568<br>P<0.0001 | -0.091<br>P<0.01 |
| GY and PH | 0.243<br>P<0.001 | 0.502<br>P<0.001 | 0.035<br>NS | - | 0.549<br>P<0.01 | 0.514<br>P<0.01 |

NS non-significant at P<0.05

Supplementary Table S5 Correlation between traits and population structure from multiple regression analysis

| Traits | Environments | | | | | |
|---|---|---|---|---|---|---|
| | Bed 5IR | BedZT 5IR | Flat 5IR | Bed drip | Bed 2IR | Bed heat |
| DH | 4.9* | 5.0 | 6.6 | 7.0 | 5.9 | 4.1 |
| | 0.007** | 0.001 | 0.05 | 0.008 | 0.001 | 0.009 |
| GY | 7.0 | 7.5 | 6.1 | 9.2 | 7.6 | 6.3 |
| | 0.001 | 0.005 | 0.001 | 0.01 | 0.01 | 0.006 |
| PH | 15.8 | 17.3 | 16.0 | - | 16.4 | 14.2 |
| | 0.001 | 0.001 | 0.05 | | 0.006 | 0.05 |

*$R^2$ in %

** *P* value

Supplementary Table S6 Marker-trait associations for grain yield under different environments using MLM and MLMM analyses

| SNP code | Clone ID | Chromosome | Position (cM) | Bed 5IR | BedZT 5IR | Flat 5IR | Bed drip | Bed 2IR | Bed heat | DSI | HSI | Comment* |
|---|---|---|---|---|---|---|---|---|---|---|---|---|
| M3989[a] | 1195316 | 1B | 137.71 | | | | | | -0.116 | | 0.030 | Novel |
| M4026[b] | 1404300 | 1B | 202.01 | | | | | | -0.112 | | 0.035 | Novel |
| M7629[b] | 1199138 | 2A | 18.22 | 0.099 | | **0.139** | | | | | | Known[15] |
| M5994[a] | 1024649 | 2A | 19.21 | | | **0.109** | | | | | | |
| M2018[a] | 1013903 | 2B | 140.01 | -0.135 | | | | | | | | Known[15] |
| M2386[b] | 988728 | 2B | 22.41 | | | | **0.141** | | | **-0.025** | | Novel |
| M1021[b] | 1121362 | 2B | 147.53 | | -0.183 | | | **-0.146** | -0.223 | | | Known[17] |
| M8374[a] | 2309600 | 2B | 147.53 | | | | | | **-0.194** | | **0.060** | |
| M5705[a] | 1386000 | 2B | 150.25 | | -0.115 | | **0.148** | | | **-0.025** | | |
| M3150[b] | 996372 | 3A | 137.76 | | **-0.124** | **0.172** | **-0.141** | **-0.183** | **-0.114** | 0.023 | | Known[17, 32, 33] |
| M7482[b] | 2265707 | 3A | 242.72 | | 0.089 | **0.156** | | **0.129** | | | | Novel |
| M4479[a] | 1095926 | 3B | 94.94 | 0.099 | | | | | | | | Known[13, 34] |
| M4718[a] | 1228289 | 3B | 297.56 | | | | **-0.187** | | | **0.035** | | Known[15, 34] |
| M7810[a] | 1098221 | 3B | 297.56 | | | | 0.101 | **0.119** | | **-0.017** | | |
| M4418[b] | 1003553 | 4A | 177.34 | 0.086 | | | **-0.124** | | | **0.025** | | Known[17] |
| M9969[a] | 2266806 | 4A | 177.34 | | | | | **-0.121** | | | | |
| M2195[b] | 992502 | 4A | 233.75 | | **0.129** | | | 0.063 | **0.069** | | | Known[13, 15, 17, 35] |
| M8365[b] | 1192194 | 4A | 53.74 | | **0.148** | | | 0.138 | **0.136** | | | Novel |
| M6564[b] | 2255707 | 4A | 48.95 | | **0.180** | | | | **0.199** | | **-0.037** | |
| M612[b] | 977534 | 4A | 48.95 | | 0.120 | | | **0.141** | 0.126 | | | |
| M2118[a] | 1125898 | 5B | 156.57 | 0.132 | | | | | | | | Known[13, 15] |
| M5964[b] | 1255792 | 5B | 153.69 | | **-0.101** | **-0.102** | | **-0.108** | | | | |
| *Vrn-B1*[b] | | 5B | 165.80 | | | | -0.157 | -0.231 | | 0.027 | | |
| M3762[a] | 1109166 | 5B | 55.28 | | | **0.135** | | | | | | Known[15] |
| M3487[a] | 1001679 | 5B | 55.28 | | | | | -0.094 | | | | |
| M306[a] | 976877 | 5B | 82.46 | | | | **-0.138** | | | **0.024** | | Novel |

Supplementary Table S6 (contd) Marker-trait associations for grain yield under different environments using MLM and MLMM analyses

| SNP code | Clone ID | Chromosome | Position (cM) | Bed 5IR | BedZT 5IR | Flat 5IR | Bed drip | Bed 2IR | Bed heat | DSI | HSI | Comment* |
|---|---|---|---|---|---|---|---|---|---|---|---|---|
| <u>M6272</u> [b] | 2266544 | 5B | 82.77 | | **-0.121** | | **-0.089** | | | | | |
| M4010 [a] | 1205840 | 6A | 188.83 | | | | | | -0.179 | | | Known[13, 15] |
| M7096 [a] | 2256226 | 6A | 165.94 | | | | | | 0.160 | | -0.052 | Novel |
| M4311 [a] | 1200675 | 6B | 5.21 | -0.172 | | | | | | | | Known[13] |
| <u>M8490</u> [a] | 2267049 | 7A | 170.91 | | 0.095 | | 0.074 | 0.068 | | | | Known[13, 15] |
| <u>M4129</u> [a] | 1194387 | 7A | 173.30 | | | | | | **0.135** | | **-0.050** | Known[13, 15] |
| M7175 [b] | 1092695 | 7B | 202.20 | | 0.143 | | 0.106 | | 0.150 | | -0.030 | Novel |
| M1441 [b] | 989685 | 7B | 112.24 | | | **-0.136** | -0.076 | | | | | Novel |
| M845 [a] | 1072804 | 7B | 188.81 | | | | -0.101 | | | 0.017 | | Known[15, 38] |
| M7896 [a] | 1119182 | 7B | 242.06 | | | | | | **-0.192** | | **0.044** | Known[15] |
| M6533 [a] | 2242441 | 7D | 224.31 | **0.184** | | | | | | | | Known |
| M4711 [a] | 1111392 | 7D | 213.42 | **0.181** | | | | | | | | Known |
| *Ppd-D1* Ciano type [b] | | | | | | | -0.247 | | | 0.047 | | |
| *Ppd-D1* Mercia type [b] | | | | | | | 0.222 | | | -0.045 | | |

For each marker, chromosome, position, and allelic effects (All Eff) are presented. Underlined markers represent same genomic region on a chromosome. Values after marker positions represent allelic effects of the markers under different environments. Values in bold represent allelic effects of the markers that survived covariate analysis with DH genes.

[a, b] signs in superscript indicate markers identified only in MLM analysis (superscript a) and in both MLM and MLMM analyses (superscript b)

* Markers identified here are categorized into novel and known genomic regions. Since the average LD decay across genome was 5cM, the markers were identified within 5cM of known QTL are reported as known and the corresponding reference has been superscripted. For markers belonging to same genomic region/QTL, the comment has been put only on the first marker reported from that genomic region.

Supplementary Table S7 Marker-trait associations identified for grain yield (GY) and Yield Stability Coefficient (Yld St) exclusively in multi-locus mixed-model analysis

| SNP code | Clone ID | Chr | Pos (cM) | Yld St *P* value (EBIC) | GY | | | | | | |
|---|---|---|---|---|---|---|---|---|---|---|---|
| | | | | | Flat 5IR | Bed ZT 5IR | Bed drip | Bed heat | DSI | HSI | Comment |
| M4782 | 992333 | 1B | 158.78 | | | | | 1.583e-07 | | 1.631e-08 | Novel |
| M2337 | 1220556 | 3A | 203.85 | | | | | 2.538e-06 | | 2.538e-06 | Novel |
| M3962 | 1035788 | 3B | 159.02 | 5.400e-06 | | 7.714e-05 | | | | | Known[13] |
| M2683 | 1217870 | 3B | 175.53 | | | | 1.288e-05 | | 1.461e-04 | | Novel |
| M9699 | 2288336 | 7B | 194.87 | 2.802e-05 | | 3.688e-05 | | | | | Known[15, 38] |
| M7958 | 985691 | 7B | 195.73 | | 1.456e-04 | | | | | | Known[15, 38] |

Underlined markers belong to same genomic region/QTL

Supplementary Table S8 GBS sequences of markers associated with yield stability coefficient

<table>
<tr><th>Marker ID</th><th>SNP ID</th><th>Sequence</th></tr>
<tr><td>M1021</td><td>1121362|F|0--17:G>A</td><td>TGCAGTGCAACACACACGCGGCCACGCATGCAGCAGGCATTGCGACAAAACATCACGCTCGCGGCCTGC</td></tr>
<tr><td>M5705</td><td>1386000|F|0--27:C>G</td><td>TGCAGCAGCTCTACTTCTGCTCATCTCCAAAGGAAGAAAGTACTACTTCTGCGCCTCCGCTGCATCAAC</td></tr>
<tr><td>M3150</td><td>996372|F|0--17:T>C</td><td>TGCAGGTGGCCCCACTTTCCACTTCGTATTGGATTCCTTCTGCACTTTTCACCATTTGTCAGCTATATA</td></tr>
<tr><td>M7482</td><td>2265707|F|0--17:G>C</td><td>TGCAGCAGGCACAACAGGTTGGAACATGGGCGCCAGAGCCGCTCCATCCGTCCTCGATCGGTCACACTT</td></tr>
<tr><td>M7810</td><td>1098221|F|0--6:A>G</td><td>TGCAGCAGAAGCAGAGTAGGAGGATGGCGGCGGGAGTGGTGCGCCGCCGCCTCGCCCTCGCCGAGATCG</td></tr>
<tr><td>M6564</td><td>2255707|F|0--67:G>A</td><td>TGCAGCTATTTGTGCATGCTTCGTCGTCGTCGACGGTGTGAAGGCATTTTTCGACGGCTACCCCGAAGC</td></tr>
<tr><td>M612</td><td>977534|F|0--37:G>C</td><td>TGCAGCGATAAGTAGTAGTAGTACTTAGTGTTGTAATGACACTTAACTATCAACAGTAACCGAGATCGG</td></tr>
<tr><td>M8365</td><td>1192194|F|0--49:A>G</td><td>TGCAGCAGAACTCGCTAATACTACGTTACAAAAAAAAACTCTTATGAACAGGCAAAACTGTAGACCCCC</td></tr>
<tr><td>M2195</td><td>992502|F|0--6:C>A</td><td>TGCAGACTTGCCGCCATGACCTTTCTATCTACTGATTTGATTGGATGTCATGCTCGGCGGGATCCCTCG</td></tr>
<tr><td>M5964</td><td>1255792|F|0--52:C>G</td><td>TGCAGCTCGGCCAGACGCCGCTGGTGGTGGTGTCGTCCAAGGAGACGGCGCGCCTGGTGCTCAAGACCC</td></tr>
<tr><td>M6272</td><td>2266544|F|0--57:G>C</td><td>TGCAGCCGAGAAAGCCAGGGTCACATCAAAGTTTGTGTAACGTACTCAGGAAATCCAGGTACTCGAGCT</td></tr>
<tr><td>M306</td><td>976877|F|0--25:G>A</td><td>TGCAGTGATGAATAAACAGAGAGGCGATTTTGGATCCCTTATGATTTGCTCGTACTTTGCTAATCTCTC</td></tr>
<tr><td>M8490</td><td>2267049|F|0--35:T>C</td><td>TGCAGTACAAATCTCCTCCACTTCGTGATAACATGTGTTAAAAACAAGAAAGATCCGTCGAGCAGGCAG</td></tr>
<tr><td>M7175</td><td>1092695|F|0--44:G>C</td><td>TGCAGCGGAGCATCCTCGCGCTGAACGGAAAGGCTGCTTAAAACGCTCGTGCAGTCCAGTTGCAGCGAG</td></tr>
<tr><td>M1441</td><td>989685|F|0--12:A>G</td><td>TGCAGTGTCTCCACGCGACCCACCCCGATGCAGGCCGCGTGAAGGCCGCCGTACTGGGACGCCAGGGAC</td></tr>
</table>

Supplementary Table S9 Marker-trait associations for days to heading under different environments using MLM and MLMM analyses

| SNP code | Clone ID | Chromosome | Position (cM) | Bed 5IR | BedZT 5IR | Flat 5IR | Bed drip | Bed 2IR | Bed heat |
|---|---|---|---|---|---|---|---|---|---|
| M3150 [b] | 996372 | 3A | 137.76 | 1.74 | 1.99 | 2.10 | | 1.77 | 1.04 |
| M7482 [a] | 2265707 | 3A | 242.72 | | | -1.49 | | | |
| M6882 [a] | 2276075 | 3A | 210.94 | | | -1.27 | | | |
| M6946 [a] | 1164647 | 3A | 55.90 | | | | -1.12 | -0.95 | |
| M9867 [a] | 1053955 | 3B | 94.98 | | | | | -0.97 | |
| M9969 [b] | 2266806 | 4A | 177.34 | 0.91 | 1.42 | 1.16 | | 1.08 | |
| M262 [a] | 1071810 | 4A | 178.31 | | | | | 1.21 | |
| M7022 [a] | 1696122 | 4B | 86.87 | | -2.16 | | | | |
| *Vrn-B1a* [b] | | 5B | 165.80 | 2.19 | 2.20 | 2.26 | 3.59 | 3.19 | 1.37 |
| M6414 [a] | 2283173 | 5B | 66.66 | | | -1.81 | | | |
| M306 [b] | 976877 | 5B | 82.46 | | | | 1.19 | | 0.82 |
| M6460 [b] | 2267368 | 5B | 152.32 | | | | | -1.23 | -0.81 |
| M2222 [a] | 1093026 | 6A | 122.86 | 1.02 | | | | 1.05 | |
| M5183 [b] | 1050270 | 6A | 122.86 | 1.01 | | | | 1.17 | |
| M4216 [b] | 1082949 | 6A | 168.41 | -1.17 | -1.62 | | -1.60 | -1.34 | -0.96 |
| M3870 [a] | 997146 | 7B | 242.25 | | 1.08 | 1.09 | | 0.92 | |
| *Ppd-D1a* (Ciano type) [b] | | | | | | | 2.79 | | 1.14 |
| *Ppd-D1* (Mercia type) [b] | | | | | | | -2.43 | | -1.26 |
| *Ppd-B1a* [b] | | | | | | | | -0.75 | |

Underlined markers represent same genomic region on a chromosome. Values after marker positions represent allelic effects of the markers under different environments

[a, b] signs in superscript indicate markers identified only in MLM analysis (superscript a) and in both MLM and MLMM analyses (superscript b)

Supplementary Table S10 Marker-trait associations for plant height under different environments using MLM and MLMM analyses

| SNP code | Clone ID | Chromosome | Position (cM) | Bed 5IR | BedZT 5IR | Flat 5IR | Bed 2IR | Bed heat |
|---|---|---|---|---|---|---|---|---|
| <u>M3183</u>[b] | 1125541 | 2B | 117.77 | | | | | 1.788 |
| <u>M245</u>[a] | 978657 | 2B | 118.92 | | | | | 2.026 |
| <u>M3657</u>[a] | 1021699 | 2B | 116.12 | | | | | 1.281 |
| <u>M2120</u>[a] | 1128199 | 2B | 114.33 | | | | | 1.427 |
| M6270[a] | 2262002 | 3B | 142.00 | -2.853 | | | | |
| M6607[b] | 2264129 | 3B | 94.94 | 1.378 | | | | |
| M5976[a] | 1011847 | 5B | 49.46 | | | -1.119 | | |
| *Rht-2*[b] | | | | 3.017 | | | 4.087 | 3.424 |
| *Rht-1*[b] | | | | -2.348 | -2.500 | -2.287 | -3.698 | |

Underlined markers represent same genomic region on a chromosome. Values after marker positions represent allelic effects of the markers under different environments

[a, b] signs in superscript indicate markers identified only in MLM analysis (superscript a) and in both MLM and MLMM analyses (superscript b)

Supplementary Table S11 Map positions of major genes on consensus map

| Gene | Chromosome | Position (cM) |
|---|---|---|
| *Ppd-A1* | 2A | 51.42 |
| *Ppd-B1* | 2B | 60.34 |
| *Rht-B1* | 4B | 37.75 |
| *Vrn-A1* | 5A | 168.64 |
| *Vrn-B1* | 5B | 165.80 |
| *Vrn-D3* | 7D | 109.43 |

Supplementary Table S12 Marker-trait associations identified for days to heading (DH) exclusively in multi-locus mixed-model analysis. For PH none of the markers was exclusive to MLMM analysis

| Trait | SNP code/gene allele | Clone ID | Chromosome | Position (cM) | *P* value | | | | | |
|---|---|---|---|---|---|---|---|---|---|---|
| | | | | | Bed 5IR | Flat 5IR | Bed ZT 5IR | Bed drip | Bed 2IR | Bed heat |
| DH | M6121 | 990721 | 2D | 36.14 | | 9.478e-05 | 3.107e-07 | | | |
| | *Vrn-D3* | - | 7D | 109.43 | 1.056e-07 | 3.107e-07 | 4.560e-08 | 2.239e-06 | 2.649e-06 | 2.571e-07 |

Supplementary Table S13 Epistatic interactions for grain yield and yield stability coefficient among markers with and without main effects under different treatments

| Treatment | Locus combination | Markers and gene alleles involved in epistasis | Percentage variation explained by interaction (%) |
|---|---|---|---|
| Bed 5IR | 2 | M1644(AA) M2145(AA) | 3.1 |
| | 2 | M2145(AA) M4418(BB)* | 3.2 |
| | 2 | M1281(BB) M4418(AA)* | 3.2 |
| | 3 | M9211(BB) M1401(AA) M7283(BB) | 3.0 |
| | 3 | M1401(AA) M7283(BB) M8358(AA) | 3.0 |
| | 3 | M330(AA) M1644(AA) M2145(AA) | 3.1 |
| | 3 | M330(AA) M2145(AA) M4418(BB)* | 3.2 |
| | 3 | M1644(AA) M2145(AA) M4418(BB)* | 3.2 |
| | 3 | M6411(AA) M2145(AA) M4418(BB)* | 2.9 |
| | 3 | M2138(BB) M1281(BB) M4418(AA)* | 3.1 |
| | 3 | M5437(BB) M1281(BB) M4418(AA)* | 3.5 |
| | 3 | M1487(AA) M1281(BB) M4418(AA)* | 2.9 |
| Bed ZT 5IR | 2 | M2403(AA) M2716(AA) | 3.5 |
| | 2 | M3159(BB) M2716(AA) | 3.7 |
| | 2 | M3598(BB) M2716(AA) | 3.6 |
| | 2 | M2716(BB) M1436(AA) | 3.6 |
| | 2 | M2716(AA) M2195(AA)* | 3.3 |
| | 3 | M332(AA) M1436(AA) M3526(BB) | 3.6 |
| | 3 | M6564(BB)* M5011(AA) M2716(BB) | 3.6 |
| | 3 | M5011(AA) M2716(BB) M1436(AA) | 4.0 |
| | 3 | M5011(AA) M1436(AA) M3526(BB) | 3.6 |
| | 3 | M5011(AA) M3526(BB) M8365(BB)* | 3.6 |
| | 3 | M3598(BB) M2716(AA) M4759(BB) | 3.5 |
| | 3 | M3598(BB) M2716(AA) M2195(AA)* | 3.5 |
| | 3 | M8728(BB) M3526(BB) M8365(BB)* | 4.2 |
| Flat 5IR | 2 | - | - |
| | 3 | - | - |
| Bed drip | 2 | M7130(BB) M3125(AA) | 3.6 |
| | 2 | M42(AA) M3125(AA) | 3.5 |
| | 2 | M6636(AA) M3125(AA) | 3.5 |
| | 2 | M3125(AA) M4718(BB)* | 3.4 |
| | 3 | M42(AA) M3050(BB) M3125(AA) | 4.0 |
| | 3 | M42(AA) M7804(BB) M3125(AA) | 4.4 |
| | 3 | M42(AA) M3125(AA) M4718(BB)* | 4.9 |
| Bed 2IR | 2 | M4407(AA) M612(AA)* | 2.5 |
| | 3 | M4104(BB) M4407(AA) M612(AA)* | 2.5 |
| | 3 | M4538(BB) M4407(AA) M612(AA)* | 2.6 |
| | 3 | M750(AA) M4407(AA) M612(AA)* | 2.5 |
| | 3 | M6895(BB) M4407(AA) M612(AA)* | 2.6 |
| | 3 | M4407(AA) M7482(BB) M612(AA)* | 2.6 |
| | 3 | M4407(AA) M1021(BB)* M612(AA)* | 2.4 |
| | 3 | M4407(AA) M612(AA)* M3150(BB)*s | 2.4 |

| Treatment | Locus combination | Markers and gene alleles involved in epistasis | Percentage variation explained by interaction (%) |
|---|---|---|---|
| Bed heat | 2 | M1659(AA) M692(BB) | 3.1 |
| | 2 | M1659(AA) M4129(BB)* | 3.2 |
| | 2 | M612(AA)* M4129(BB)* | 3.2 |
| | 2 | M692(BB) M4782(AA) | 3.0 |
| | 2 | M4782(AA) M2337(BB) | 3.0 |
| | 2 | M4782(AA) M4129(BB)* | 3.1 |
| | 2 | M4129(BB)* M6564(BB)* | 3.2 |
| | 3 | M1659(AA) M4787(BB) M4129(BB)* | 3.0 |
| | 3 | M1659(AA) M692(BB) M4782(AA) | 3.0 |
| | 3 | M1659(AA) M692(BB) M2337(BB) | 3.1 |
| | 3 | M1659(AA) M4782(AA) M4129(BB)* | 2.9 |
| | 3 | M1659(AA) M3989(AA) M4129(BB)* | 3.0 |
| | 3 | M4787(BB) M612(AA)* M4129(BB)* | 3.0 |
| | 3 | M10214(BB) M612(AA)* M692(BB) | 4.1 |
| | 3 | M612(AA)* M4782(AA) M4129(BB)* | 3.1 |
| | 3 | M612(AA)* M3989(AA) M4129(BB)* | 3.0 |
| | 3 | M692(BB) M4782(AA) M2337(BB) | 2.9 |
| | 3 | M4782(AA) M4129(BB)* M6564(BB)* | 3.1 |
| Yield stability coefficient | 2 | M1436(AA) M3125(BB) | 8.1 |
| | 2 | M5456(AA) M3125(AA) | 8.2 |
| | 2 | M6957(AA) M3125(BB) | 8.0 |
| | 2 | M6706(BB) M3125(AA) | 8.2 |
| | 2 | M2264(AA) M3125(AA) | 8.8 |
| | 2 | M3125(BB) M612(AA)* | 8.3 |
| | 3 | M1436(AA) M6957(AA) M3125(BB) | 9.5 |
| | 3 | M1436(AA) M6957(AA) M2716(BB) | 8.9 |
| | 3 | M6957(AA) M2337(BB) M3125(BB) | 9.4 |
| | 3 | M6957(AA) M3125(BB) M612(AA)* | 9.2 |
| | 3 | M6957(AA) M3125(BB) M6564(BB)* | 8.9 |

The underlined marker indicates the main epistatic locus identified and asterisk sign indicates markers with main effects

Supplementary Table S14 Epistatic interactions for grain yield and yield stability coefficient among markers without main effects

| Treatment | Locus combination | Marker alleles involved in epistasis | Percentage variation explained by interaction (%) |
|---|---|---|---|
| Bed 5IR | 2 | M1401(AA) M3204(BB) | 3.2 |
| | 2 | M1401(AA) M3303(BB) | 3.1 |
| | 2 | M1401(AA) M1281(BB) | 3.1 |
| | 2 | M1644(AA) M2145(AA) | 3.3 |
| | 3 | M9211(BB) M1401(AA) M7283(BB) | 3.0 |
| | 3 | M1401(AA) M7283(BB) M8358(AA) | 3.0 |
| | 3 | M330(AA) M1644(AA) M2145(AA) | 3.1 |
| Bed ZT 5IR | 2 | M2403(AA) M2716(AA) | 3.5 |
| | 2 | M3159(BB) M2716(AA) | 3.7 |
| | 2 | M3598(BB) M2716(AA) | 3.3 |
| | 2 | M2716(BB) M1436(AA) | 3.6 |
| | 3 | M332(AA) M1436(AA) M3526(BB) | 3.6 |
| | 3 | M5011(AA) M2716(BB) M1436(AA) | 3.4 |
| | 3 | M5011(AA) M1436(AA) M3526(BB) | 3.2 |
| | 3 | M3598(BB) M2716(AA) M4759(BB) | 3.5 |
| Flat 5IR | 2 | M6636(AA) M2259(BB) | 4.1 |
| | 2 | M282(AA) M2259(BB) | 4.0 |
| | 2 | M2259(BB) M2773(AA) | 4.0 |
| | 2 | M2259(BB) M9968(BB) | 3.6 |
| | 2 | M2259(BB) M7617(BB) | 3.8 |
| | 2 | M2259(BB) M1757(BB) | 4.0 |

| Treatment | Locus combination | Marker alleles involved in epistasis | Percentage variation explained by interaction (%) |
| --- | --- | --- | --- |
| Flat 5IR | 2 | M2259(BB) M4169(BB) | 3.8 |
| | 3 | M6636(AA) M282(AA) M2259(BB) | 4.1 |
| | 3 | M6636(AA) M2259(BB) M2808(AA) | 4.2 |
| | 3 | M6636(AA) M2259(BB) M1069(AA) | 4.0 |
| | 3 | M282(AA) M2259(BB) M2209(BB) | 4.0 |
| Bed drip | 2 | M7130(BB) M3125(AA) | 3.5 |
| | 2 | M42(AA) M3125(AA) | 3.5 |
| | 2 | M3050(BB) M3125(AA) | 3.4 |
| | 2 | M7804(BB) M3125(AA) | 3.4 |
| | 2 | M6636(AA) M3125(AA) | 3.5 |
| | 3 | M7130(BB) M42(AA) M3125(AA) | 3.5 |
| | 3 | M7130(BB) M1786(BB) M3125(AA) | 3.5 |
| | 3 | M7130(BB) M3125(AA) M4962(BB) | 3.0 |
| | 3 | M42(AA) M1786(BB) M3125(AA) | 3.4 |
| | 3 | M42(AA) M3050(BB) M3125(AA) | 3.4 |
| | 3 | M3050(BB) M6636(AA) M3125(AA) | 3.4 |
| Bed 2IR | 2 | M2493(AA) M4407(BB) | 2.5 |
| | 3 | M2493(AA) M6528(BB) M4407(BB) | 2.4 |
| | 3 | M2493(AA) M4607(BB) M4407(BB) | 2.5 |
| | 3 | M2493(AA) M262(BB) M4407(BB) | 2.4 |
| | 3 | M2493(AA) M1013(BB) M4407(BB) | 2.1 |

| Treatment | Locus combination | Marker alleles involved in epistasis | Percentage variation explained by interaction (%) |
|---|---|---|---|
| Bed heat | 2 | M1659(AA) M692(BB) | 3.1 |
| | 2 | M692(BB) M4782(AA) | 3.0 |
| | 2 | M4782(AA) M2337(BB) | 3.0 |
| | 3 | M692(BB) M4782(AA) M2337(BB) | 3.0 |
| Yield stability coefficient | 2 | M1436(AA) M3125(BB) | 9.0 |
| | 2 | M5456(AA) M3125(AA) | 9.2 |
| | 2 | M6957(AA) M3125(BB) | 8.9 |
| | 2 | M6706(BB) M3125(AA) | 8.2 |
| | 2 | M2264(AA) M3125(AA) | 8.8 |
| | 3 | M1436(AA) M6957(AA) M3125(BB) | 8.7 |
| | 3 | M1436(AA) M6957(AA) M2716(BB) | 8.9 |
| | 3 | M6957(AA) M2337(BB) M3125(BB) | 9.4 |

The underlined marker indicates the main epistatic locus

Supplementary Table S15 Lines identified with best allelic combination of the four significant markers identified by forward stepwise regression analysis

| GID | M3125 | M6564 | M3150 | M1021 | $P_i$ | Mean GY (tons/ha) | DSI | HSI |
|---|---|---|---|---|---|---|---|---|
| 6177498 | A | A | B | B | 1.96 | 4.7 | 1.00 | 0.98 |
| 6177599 | A | A | B | B | 1.83 | 4.9 | 0.99 | 0.97 |
| 6177657 | A | A | B | B | 1.29 | 5.1 | 0.91 | 0.77 |
| 6177659 | A | A | B | B | 1.18 | 5.2 | 0.94 | 0.98 |
| 6177665 | A | A | B | B | 1.31 | 5.2 | 0.96 | 1.00 |
| 6178397 | A | A | B | B | 1.18 | 5.2 | 0.96 | 0.85 |
| 6178398 | A | A | B | B | 1.13 | 5.3 | 0.84 | 0.97 |
| 6178403 | A | A | B | B | 1.09 | 5.2 | 0.98 | 1.00 |
| 6178404 | A | A | B | B | 1.16 | 5.2 | 0.98 | 1.00 |
| 6178527 | A | A | B | B | 1.13 | 5.2 | 0.91 | 0.90 |
| 6178528 | A | A | B | B | 1.24 | 5.2 | 0.90 | 0.71 |
| 6178541 | A | A | B | B | 1.42 | 5.0 | 0.93 | 0.86 |
| 6178556 | A | A | B | B | 1.26 | 5.2 | 0.80 | 1.00 |
| 6179132 | A | A | B | B | 0.67 | 5.6 | 0.92 | 1.00 |
| 6179216 | A | A | B | B | 0.65 | 5.6 | 1.00 | 0.95 |
| 6179220 | A | A | B | B | 0.66 | 5.6 | 1.00 | 0.96 |
| 6179510 | A | A | B | B | 1.15 | 5.2 | 0.98 | 0.97 |
| 6179543 | A | A | B | B | 1.00 | 5.3 | 0.95 | 0.91 |
| 6181747 | A | A | B | B | 1.48 | 5.0 | 1.00 | 0.98 |
| 6181755 | A | A | B | B | 1.68 | 4.4 | 0.86 | 0.98 |
| 6181760 | A | A | B | B | 1.55 | 5.0 | 0.90 | 1.00 |

$P_i$ Yield stability coefficient

DSI Drought susceptibility index

HSI Heat susceptibility index

Supplementary Table S16 *In silico* candidate gene analysis of significant markers identified by stepwise regression analysis

| SNP code | Allele | Blast Hit Accession | Consequence | Gene description | Interpro-ID (Description/Domain) |
|---|---|---|---|---|---|
| M1021 | C | AT4G27590.2 | 3’UTRvariant | Heavy metal transport/detoxification superfamily protein LENGTH=332 | IPR006121 (Heavy metal-associated domain, HMA) |
| M3150 | T | AT5G18750.1 | Downstream gene variant | DNAJ heat shock N-terminal domain-containing protein LENGTH=884 | IPR024593 (Domain of unknown function DUF3444) |

Supplementary Table S17 Allele specific SNP and STS markers for major genes controlling phenology and plant height used in the present study

| Gene-specific allele | Marker type* | Function/Effect | Source/Reference |
|---|---|---|---|
| *Vrn-A1a* | SNP | Spring growth habit | CerealsDB; Yan et al. (2004) |
| *Vrn-A1b* | SNP | Spring growth habit | CerealsDB; Yan et al. (2004) |
| *Vrn-A1b* (Jagger type) | SNP | Short vernalizing | CerealsDB; Chen et al. (2009) |
| *Vrn-A1v/w* | CAPS | Winter growth habit | ------------- |
| *Vrn-B1a* | STS | Spring growth habit | MASWHEAT; Fu et al. 2005 |
| *Vrn-B1b* | STS | Spring growth habit | Santra et al. (2009) |
| *Vrn-D1* | SNP | Spring growth habit | CerealsDB; |
| *Vrn-D3* | SNP | Spring growth habit | Gina Brown, USDA (Pers. commun.); (Chen et al. 2010) |
| *Ppd-A1a* (GS-100 type) | SNP | Photoperiod insensitivity | CerealsDB; (Wilhelm et al. 2009) |
| *Ppd-A1a* (GS-105 type) | SNP | Photoperiod insensitivity | CerealsDB; (Wilhelm et al. 2009) |
| *Ppd-A1* (Capelle-Desprez deletion) null allele | SNP | Photoperiod insensitivity | CerealsDB; (Beales et al. 2007) |
| *Ppd-A1b* (Intron-5 insertion) | SNP | Photoperiod sensitivity | Gina Brown, USDA (Pers. commun.) |
| *Ppd_B1a* (Chinese Spring retrotransposon exon 7) | SNP | Photoperiod insensitivity | CerealsDB; (Beales et al. 2007) |
| *Ppd-B1a* (Chinese Spring exon 3) | SNP | Photoperiod insensitivity | Gina Brown, USDA (Pers. commun.) |
| *Ppd-B1b* (Chinese Spring Line Element SNP4) | SNP | Photoperiod insensitivity | Gina Brown, USDA (Pers. commun.) |
| *Ppd-B1b* (Chinese Spring SNP5) | SNP | Photoperiod insensitivity | Gina Brown, USDA (Pers. commun.) |
| *Ppd-B1a* (Sonora 64 type deletion) | SNP | Photoperiod insensitivity | Gina Brown, USDA (Pers. commun.) |
| *Ppd-D1a* (Ciano67 type deletion) | SNP | Photoperiod insensitivity | CerealsDB; (Beales et al. 2007) |
| *Ppd-D1b* (Mercia type Intron 1 insertion) | SNP | Photoperiod sensitivity | CerealsDB; (Beales et al. 2007) |
| *Ppd-D1b* (Norstar type deletion in exon 7) | SNP | Photoperiod sensitivity | CerealsDB; (Beales et al. 2007) |
| *Rht-B1* | SNP | Semi-dwarf | CerealsDB; Ellis et al (2002) |
| *Rht-D1* | SNP | Semi-dwarf | CerealsDB; Ellis et al (2002) |

* Genotyping was carried out in the CIMMYT laboratory using allele-specific SNP or sequence tagged site (STS) markers for each gene
